# Efficient continuous-flow synthesis of novel 1,2,3-triazole-substituted β-aminocyclohexanecarboxylic acid derivatives with gram-scale production

**DOI:** 10.3762/bjoc.9.172

**Published:** 2013-07-29

**Authors:** Sándor B Ötvös, Ádám Georgiádes, István M Mándity, Lóránd Kiss, Ferenc Fülöp

**Affiliations:** 1 Institute of Pharmaceutical Chemistry, University of Szeged, Eötvös u. 6, H-6720 Szeged, Hungary

**Keywords:** β-amino acids, click chemistry, continuous-flow, copper, flow chemistry, triazoles

## Abstract

The preparation of novel multi-substituted 1,2,3-triazole-modified β-aminocyclohexanecarboxylic acid derivatives in a simple and efficient continuous-flow procedure is reported. The 1,3-dipolar cycloaddition reactions were performed with copper powder as a readily accessible Cu(I) source. Initially, high reaction rates were achieved under high-pressure/high-temperature conditions. Subsequently, the reaction temperature was lowered to room temperature by the joint use of both basic and acidic additives to improve the safety of the synthesis, as azides were to be handled as unstable reactants. Scale-up experiments were also performed, which led to the achievement of gram-scale production in a safe and straightforward way. The obtained 1,2,3-triazole-substituted β-aminocyclohexanecarboxylates can be regarded as interesting precursors for drugs with possible biological effects.

## Introduction

**Figure 1:**
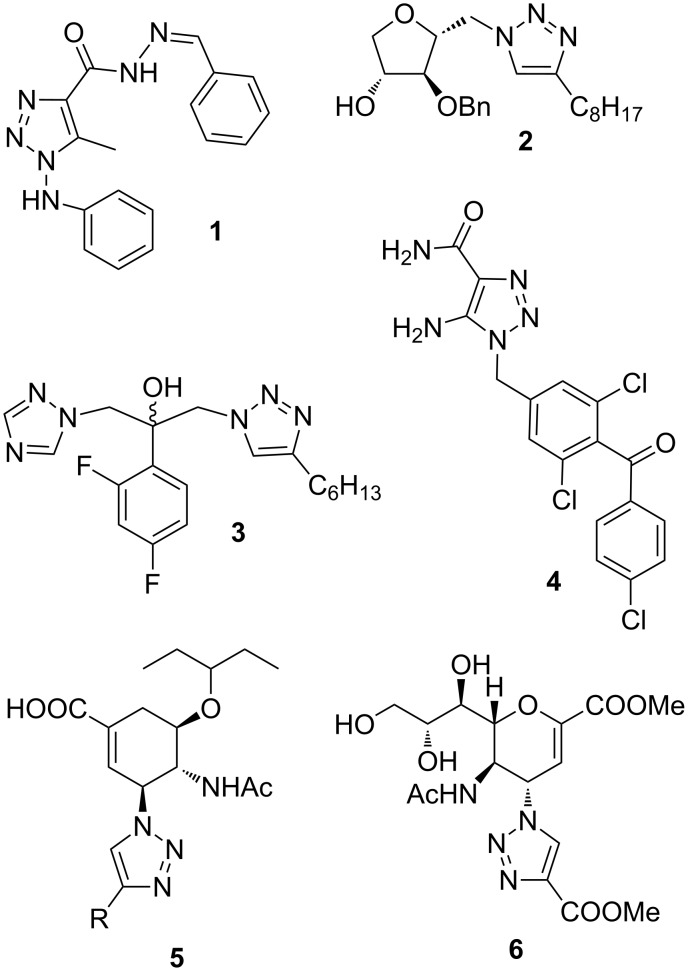
Examples of 1,2,3-triazoles with various biological activities.

In recent years, triazole-containing compounds have become potential targets for drug discovery [1–2]. A large number of 1,2,3-triazoles exhibit various biological effects [3], e.g., antiviral (**1**), antibacterial (**2**), antifungal (**3**) and anticancer (**4**) activities [4–7] (Figure 1). The 1,2,3-triazole skeleton is frequently used as a pharmacophore for the modification of known pharmaceuticals. Triazole analogues of several bioactive compounds have recently been reported. Examples are those of the well-known highly functionalized antiviral cyclic amino acid derivatives oseltamivir and zanamivir (**5** and **6** in Figure 1) [8–9]. The 1,2,3-triazole moiety is a constituent part of many modified nucleosides or carbanucleosides with antiviral, anti-HIV or cytostatic activities [10–12]. However, the scope of triazole chemistry is not confined to drug discovery. There are an increasing number of applications in numerous other areas of modern chemical sciences, such as bioconjugation [13], supramolecular chemistry, [14] and polymer sciences [15].

**Scheme 1:**
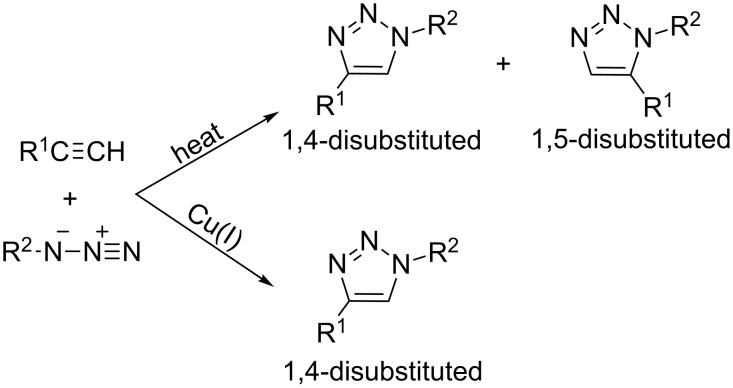
1,3-Dipolar azide–alkyne cycloadditions.

Probably the most useful and powerful procedure for the synthesis of 1,2,3-triazoles is the Huisgen 1,3-dipolar cycloaddition of organic azides with acetylenes [16]. The classical Huisgen reaction, thermally induced, gives an approximate 1:1 mixture of 1,4- and 1,5-disubstituted 1,2,3-triazole isomers (Scheme 1) [17]. However, when Cu(I) catalysis is applied, the reaction becomes regioselective, exclusively yielding the 1,4-regioisomer within a relatively short reaction time [18–20]. Recently, Cu(I)-catalyzed azide–alkyne cycloaddition (CuAAC) has become the basis of the so-called click chemistry concept due to its wide applicability and efficiency.

**Figure 2:**
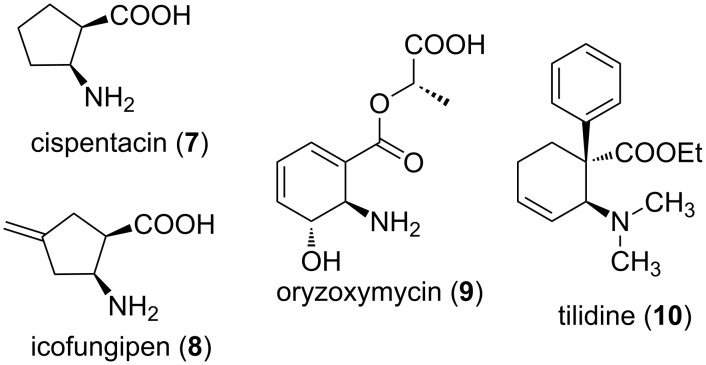
Selected bioactive alicyclic β-amino acids.

Over the past twenty years, alicyclic β-amino acids have attracted great interest among synthetic chemists, thanks to their massive pharmacological potential [21–22]. For example, cispentacin ((1*R*,2*S*)-2-aminocyclopentanecarboxylic acid, **7**) is a widely investigated naturally occurring carbocyclic β-amino acid with strong antifungal properties against *Candida* species (Figure 2) [23]. Its synthetic 4-methylene derivative icofungipen (**8**), also an antifungal agent, is now proceeding through clinical development for the oral treatment of yeast infections (Figure 2) [24]. Certain multi-substituted cyclohexane amino acid derivatives, such as oryzoxymycin (**9**) and tilidine (**10**), are also well-known bioactive agents with anticancer, antibacterial, antiviral or analgesic effects (Figure 2) [25–26]. The alicyclic β-amino acids are key intermediates for the synthesis of a series of pharmaceutically relevant products [27], such as amino esters, amino alcohols, azides and heterocycles. Moreover, they are frequently used as building blocks for the synthesis of new peptides and foldamers with possible biological effects [28].

Modern continuous-flow (CF) technologies offer many advantages over classical batch-based procedures [29–32], including efficient mixing quality [33], excellent heat and mass transfer [34], shorter reaction times [35–37], reduced reagent consumption [38–40], improved safety [41–42], and operational simplicity [43]. Furthermore, CF methodologies provide opportunities for a simple and rapid scale-up [44–45] and automation [46–47] of chemical processes. They also tend to be environmentally benign technologies [48]. In consequence of these benefits, flow chemistry-based techniques have exerted a significant impact on modern synthetic chemistry, ranging from laboratory-based experiments to industrial-scale production.

Here, we describe a safe and efficient CF synthesis of a series of novel 1,2,3-triazole-modified β-aminocyclohexanecarboxylic acid derivatives as potential biologically active compounds. Gram-scale production is also reported, which predicts a possible usefulness for the pharmaceutical industry.

## Results and Discussion

**Figure 3:**
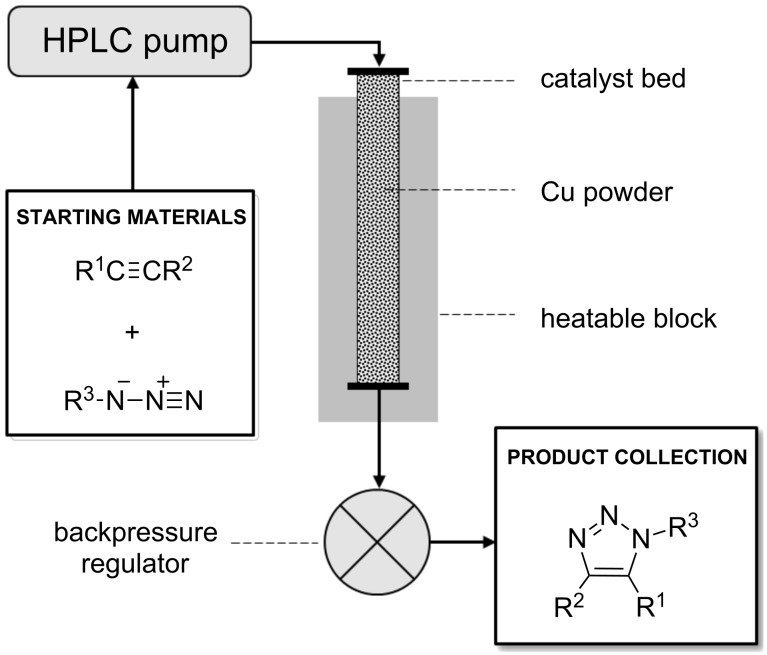
Experimental setup for the CF reactions.

Several approaches are to be found in the literature for the Cu(I)-catalysed flow synthesis of triazoles. Heterogeneous Cu(I) sources are most popular, such as copper-in-charcoal (Cu/C) [49–50], solid supported Cu(I) species [51–54], and heated copper wirings [55–58], but a homogeneous technique has also recently emerged [59]. The main driving forces behind these CF methodologies are the safety aspects associated with the handling of azides and the inherent scalability of flow processing. Moreover, when organic azides are formed in situ, operational safety can be further improved [55,57]. We envisioned that it would be simplest to make use of copper powder as a catalytic source [60]. Similarly to cases when heated rings of copper wire are employed, a copper surface acts as a source of active copper species. Copper is constantly oxidized when exposed to air, and non-self-protecting layers of different oxides, including Cu_2_O, are formed on its surface [61], which can promote CuAAC. Thus, we utilized copper powder in a stainless steel column, which served as a catalyst bed later on. The catalyst bed was placed into a stainless steel block with a Peltier heating system, which could heat the column up to 100 °C. A backpressure regulator was also integrated to ensure pressures up to 100 bar. The mixture of the reactants was pumped through the system continuously by means of an HPLC pump. This experimental setup is practical and cheap, as it does not require costly catalysts or special apparatus. At the same time this setup is safe, even with unstable reactants such as azides (Figure 3).

To maximize the CF triazole synthesis reaction rates, it appeared easiest to use high-temperature conditions initially. The application of elevated pressure in CuAAC is also beneficial, as it can promote the product formation in accordance with Le Chatelier’s principle [60] and also prevents the solvent from boiling over when high temperature is used. Thus, 100 °C and 100 bar were selected as conditions **A** for the CF synthesis. However, when azides are reacted, it is important to minimize the explosion hazard. Accordingly, we attempted to improve the rates of the reaction in the presence of additives, without the use of high temperature [60]. Amines are known to accelerate CuAAC, in particular by coordinating to catalytically active Cu(I) species and promoting their liberation from the copper matrix [62–63]. It was recently shown that the use of certain acids as additives is also beneficial, as this can further accelerate the formation of the triazole product [64–66] and also prevents the accumulation of unwanted byproducts, such as diacetylenes, bistriazoles, etc. [67]. At the same time, byproduct formation is catalysed by a base, and the joint utilization of a basic and an acidic additive is therefore favourable. This buffer system gives rise to a high reactivity in CuAAC, even at room temperature (rt), but without byproduct formation [60,67]. This system thus greatly improves the safety relative to the high-temperature conditions. The literature data led us to select *N*,*N*-diisopropylethylamine (DIEA) as a base and HOAc as an acid [67], which were used jointly as additives, each in 0.04 equivalents, at 100 bar and rt as conditions **B** [60].

As starting materials for the CF CuAAC reactions, azido-substituted β-aminocyclohexanecarboxylates **11**–**14** were prepared previously by a diastereoselective epoxidation of the corresponding 2-aminocyclohexenecarboxylates, followed by a regioselective oxirane ring opening with NaN_3_ [68]. Three different alkynes (phenylacetylene, diethyl acetylenedicarboxylate and ethynyl ferrocene) were employed as dipolarophiles to yield a library of novel 1,2,3-triazole-modified cyclic β-amino acid derivatives. Compounds **11**–**14** were racemates, the structures in Table 1 show their relative stereochemistry. The CF syntheses were carried out under both conditions **A** and **B** in order to obtain a clear comparison between the performances of the two approaches. CH_2_Cl_2_ was used as a solvent, and the starting azides were used in a concentration of 0.085 M. A higher concentration of the starting azides led to the precipitation of the triazole product and a blockage in the CF reactor. Aliquots of 2.5 mL of a reaction mixture containing 1 equivalent of the azide and 1.5 equivalents of the acetylene were pumped through the reactor in each run with a a flow rate of 0.5 mL min^–1^. At this flow rate the residence time on the catalyst bed was as low as 1.5 min and it took only 5 min of process time to pump the 2.5 mL aliquots through the system. This resulted in around 100 mg of crude product, depending on the conversion and the molecular masses of the reactants.

**Table 1:** CF synthesis of 1,2,3-triazole-substituted alicyclic β-amino acid derivatives.

Entry	Azide^a^ (1 equivalent)	Acetylene (1.5 equivalents)	Product	Yield^b^ (%)	

				**A**^c^	**B**^d^

1	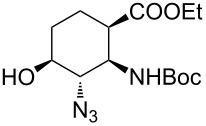 **11**	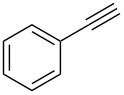	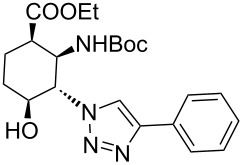 **15**	61	96
2	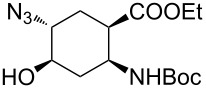 **12**	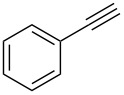	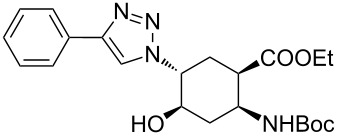 **16**	47	97
3	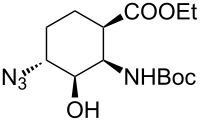 **13**	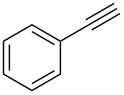	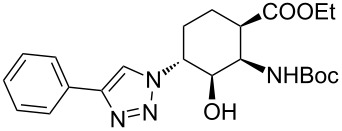 **17**	33	76 (98)^e^
4	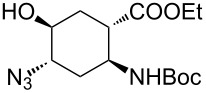 **14**	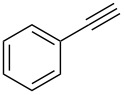	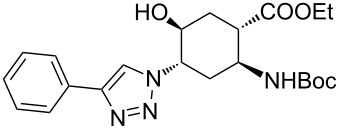 **18**	53	89 (98)^e^
5	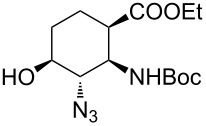 **11**	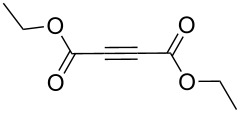	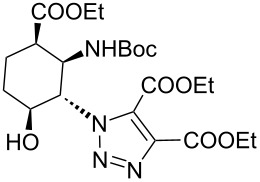 **19**	98	97
6	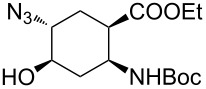 **12**	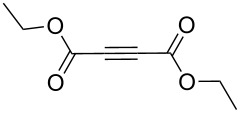	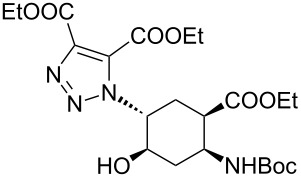 **20**	97	98
7	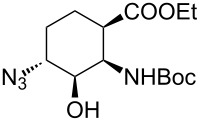 **13**	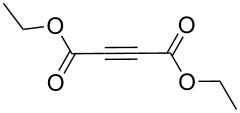	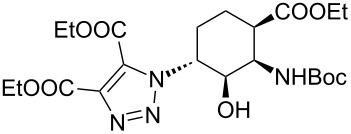 **21**	97	96
8	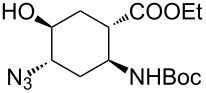 **14**	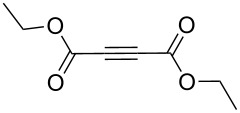	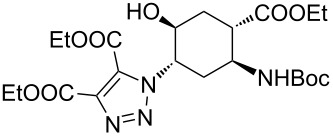 **22**	97	98
9	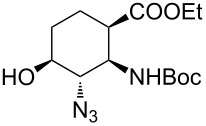 **11**	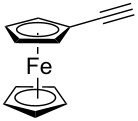	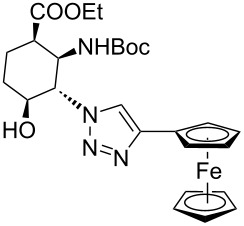 **23**	95	97
10	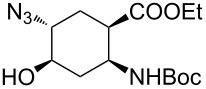 **12**	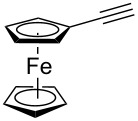	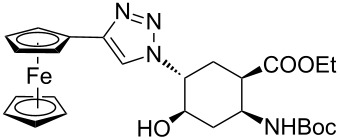 **24**	91	98
11	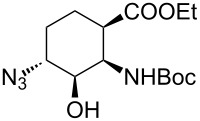 **13**	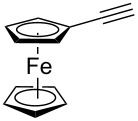	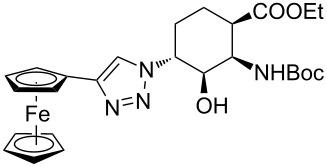 **25**	96	93
12	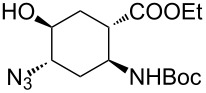 **14**	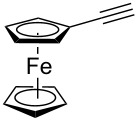	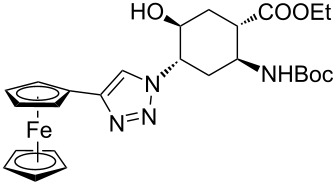 **26**	75	97

^a^*c*_azide_ = 0.085 M. ^b^Yield of isolated product. ^c^Conditions **A**: CH_2_Cl_2_ as solvent, 100 bar, 100 °C, flow rate 0.5 mL min^–1^, without any additives. ^d^Conditions **B**: CH_2_Cl_2_ as solvent, 100 bar, rt, flow rate 0.5 mL min^–1^, with 0.04 equivalents of DIEA + 0.04 equivalents of HOAc. ^e^Achieved under the following conditions: CH_2_Cl_2_ as solvent, 100 bar, 100 °C, flow rate 0.5 mL min^–1^, with 0.04 equivalents of DIEA + 0.04 equivalents of HOAc.

In the Cu(I)-catalysed reactions between phenylacetylene and the azido-substituted β-amino acid derivatives **11**–**14**, 1,4-disubstituted 1,2,3-triazole isomers (**15**–**18**) were regioselectively formed. The high-pressure/high-temperature conditions **A** led to only medium yields (Table 1, entries 1–4), but under conditions **B** the yields of triazoles **15** and **16** were excellent, and those of triazoles **17** and **18** were high (76% and 89%, respectively; Table 1, entries 1–4). When the CF reactions of azides **13** and **14** with phenylacetylene were repeated under high-pressure/high-temperature conditions with the simultaneous use of additives (100 bar, 100 °C, 0.04 equivalents each of DIEA and HOAc; further conditions were not modified), triazoles **17** and **18** were obtained in very high yields (98% in both cases; Table 1, entries 3 and 4).

1,4,5-Trisubstituted 1,2,3-triazoles are of notable importance in drug discovery. For example, several 1,2,3-triazole-4,5-dicarboxylates display significant antituberculotic activity in vitro [69]. Thus, a nonterminal alkyne, diethyl acetylenedicarboxylate, was subjected to CF CuAAC with the azido-functionalized β-amino acid derivatives **11**–**14** as reaction partners. 1,4,5-Trisubstituted 1,2,3-triazole dicarboxylates **19**–**22** were obtained in excellent yields (>96%) under both conditions **A** and **B** (Table 1, entries 5–8). In this set of CF syntheses, no significant difference was observed between the performances of the two methods.

Ferrocene-triazole conjugates play a crucial role in the labelling and detection of various systems, such as biomolecules, polymers, nanomaterials and supramolecular assemblies [70]. They also have potential applications in medicinal chemistry and drug discovery as biosensing probes, in immunoassays and in host–guest chemistry [71]. Ferrocene-substituted amino acids have been of significant importance in the investigation of the secondary structures of different peptides and foldamers [72]. Thus, conjugates of the azido-functionalized β-amino acid derivatives **11**–**14** were prepared with ethynylferrocene as a dipolarophile. Both conditions **A** and **B** afforded ferrocenyltriazoles **23**–**25** in excellent yields (>91%; Table 1, entries 9–11). However, in the case of ferrocenyltriazole **26** the high-pressure/high-temperature conditions **A** led to a yield of only 75%, whereas the use of additives at rt (conditions **B**) proved more efficient, with a yield of 97% (Table 1, entry 12). Triazoles **23**–**26** were obtained selectively as 1,4-disubstituted regioisomers.

To understand the differences between the results obtained with the three different dipholarophiles, it must be taken into account that the carboxylate groups of diethyl acetylenedicarboxylate and the aromatic system of the ferrocenyl group as ligands can probably coordinate copper from its matrix. Therefore, the concentration of the catalytically active Cu(I) species is increased as compared to the reactions with phenylacetylene [73–75]. Accordingly, the yields were usually higher in the reactions with diethyl acetylenedicarboxylate and ethynylferrocene than with phenylacetylene (Table 1, entries 5–12 versus entries 1–4). These differences can mainly be observed between the results obtained under conditions **A**. This is because the base, as an additive, evolves the same effect and improves the reactivity through the CuAAC, thus in the case of conditions **B** (the use of additives) the influence of the alkyne is practically masked.

The presence of trace amounts of copper in the chromatographically purified triazole products was determined by means of inductively coupled plasma mass spectrometry (ICP–MS). The analytical data in Table 2 show that the contents of copper impurities in the products were appropriately low, i.e., amounts of 3.9–9.1 µg g^–1^ were detected. It should be noted that the samples obtained with the joint use of DIEA + HOAc (conditions **B**) contained more copper than those obtained under conditions **A** (high-temperature/high-pressure without additives). The levels of copper contamination detected in our triazole products compare well with literature results relating to CF [50] and conventional batch experiments [76].

**Table 2:** Copper contents in the triazole products after column chromatographic purification on silica gel.

Entry	Product	Copper content (µg g^–1^)^a^	

		**A**^b^	**B**^c^

1	**15**	4.6 (±0.5)	8.4 (±0.6)
2	**16**	4.2 (±0.3)	7.7 (±0.6)
3	**17**	3.9 (±0.5)	8.0 (±0.4)
4	**18**	4.7 (±0.6)	8.2 (±0.7)
5	**19**	5.2 (±0.4)	7.9 (±0.4)
6	**20**	5.1 (±0.3)	7.5 (±0.6)
7	**21**	4.8 (±0.6)	7.7 (±0.7)
8	**22**	5.3 (±0.3)	8.2 (±0.6)
9	**23**	6.1 (±0.5)	8.6 (±0.5)
10	**24**	4.8 (±0.4)	7.7 (±0.8)
11	**25**	5.4 (±0.3)	9.1 (±0.4)
12	**26**	4.9 (±0.6)	7.8 (±0.7)

^a^Determined by ICP–MS. ^b^Conditions **A**: CH_2_Cl_2_ as solvent, 100 bar, 100 °C, flow rate 0.5 mL min^–1^, without any additives. ^c^Conditions **B**: CH_2_Cl_2_ as solvent, 100 bar, rt, flow rate 0.5 mL min^–1^, with 0.04 equivalents of DIEA + 0.04 equivalents of HOAc.

**Scheme 2:**
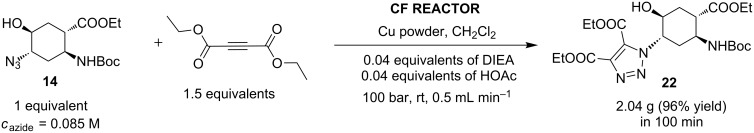
Gramm-scale CF synthesis of triazole **22** under conditions **B**.

In conventional batch-based chemistry, the scale-up of chemical reactions can be a challenge because the output depends on the batch size. The situation becomes even more complicated when unstable reactants such as azides are handled on a large scale. However, the scalability of CF procedures is a straightforward function of time and the flow rate, and the risks associated with the accumulation of hazardous species are minimized, because the solution of the reactants is eluting continuously from the active zone of the reactor [33–34,44–45,60]. The CF CuAAC between azide **14** and diethyl acetylenedicarboxylate was scaled up in a simple, safe and efficient manner to achieve gram-scale production (Scheme 2). Methods **A** and **B** proved equally efficient in the small-scale CF syntheses of triazole **22** (Table 1, entry 8). However, we performed the large-scale experiment at 100 bar and rt in the presence of the additives (conditions **B**) so as to ensure maximum safety throughout the procedure. A CH_2_Cl_2_ solution of the reaction mixture containing 1 equivalent of the azide (*c*_azide_ = 0.085 M), 1.5 equivalents of the acetylene and 0.04 equivalents of each additive was pumped continuously through the system at a flow rate of 0.5 mL min^–1^. During the whole scale-up procedure, the same portion of copper powder was used in the catalyst bed. The solution of the crude product was collected for 100 min, and after purification 2.06 g of triazole **22** was obtained, which is equivalent to a yield of 96%.

## Conclusion

Twelve highly functionalized 1,2,3-triazole-substituted β-aminocyclohexanecarboxylic acid racemates were successfully prepared in CF mode as a small library of novel compounds with possible biological effects. The CF syntheses were first performed under high-pressure/high-temperature conditions with copper powder as a readily accessible Cu(I) source. Subsequently, to moderate the harsh reaction conditions, the reaction temperature could be lowered to rt in the presence of additives. The joint use of a base and an acid dramatically improved the reactivity in the CuAAC, while it completely eliminated unwanted byproduct formation. These conditions ensured enhanced safety and typically higher yields than those attained under the harsh reaction conditions. Simple, efficient and safe gram-scale production was also implemented in a short processing time, which can be important for potential industrial applications.

## Experimental

### General Information

The reagents and materials were of the highest commercially available purity grade and were used without any further purification. Flash column chromatography was performed on Merck silica gel 60, particle sizes ranged from 63 to 200 μm, and analytical thin-layer chromatography (TLC) on Merck silica gel 60 F254 plates. Compounds were visualized with UV light or KMnO_4_. ^1^H and ^13^C NMR spectra were recorded on a Bruker Avance DRX 500 spectrometer, in CDCl_3_ as a solvent, with TMS as internal standard, and at 500.1 and 125.0 MHz, respectively. Microanalyses were performed on a Perkin-Elmer 2400 elemental analyser.

### Determination of the copper contents of the triazole products

Copper concentrations were determined by ICP–MS by using an Agilent 7700x instrument equipped with a collision cell. The determination was carried out on the isotope ^63^Cu, with He as collision gas. The standard solutions for external calibration were prepared from a stock solution (Certipur, Merck) by dilution with doubly deionized water (Millipore MillQ, Merck). All glassware and plastic utensils used during the determination were precleaned by soaking in solutions of trace-metal-grade nitric acid and hydrochloric acid (Suprapur, Merck), followed by rinsing with copious amounts of doubly deionized water.

### General procedure for the CF reactions

An H-Cube^®^ system was used as a CF reactor in the “no H_2_” mode. For the CF reactions, the catalyst bed (internal dimensions: 70 mm × 4 mm) was filled with ~900 mg of copper powder with an average particle size of 200 µm. 70 mg (0.21 mmol, 1 equivalent) of the corresponding azide and 0.32 mmol (1.5 equivalents) of the alkyne, and (only in method **B**) 1.5 µL (0.0084 mmol, 0.04 equivalents) of DIEA and 0.5 µL (0.0084 mmol, 0.04 equivalents) of HOAc were dissolved in 2.5 mL of CH_2_Cl_2_. The solution was homogenized by sonication, and then pumped through the CF reactor under the appropriate conditions. Between two reactions in the CF reactor, the catalyst bed was washed at rt for 5 min with CH_2_Cl_2_ at a flow rate of 1 mL min^–1^. The crude product was checked by TLC with a mixture of *n*-hexane/EtOAc as an eluent, and the solvent was next evaporated off under vacuum. Column chromatographic purification was carried out on silica gel with a mixture of *n*-hexane/EtOAc as an eluent. The 1,2,3-triazole-modified compounds were characterized by elemental analysis and NMR experiments. For detailed analytical data see Supporting Information File 1.

### Measurement of the residence time on the catalyst bed

To determine the residence time, a CH_2_Cl_2_ solution of a blue ink was pumped through the catalyst bed. The time that elapsed between the first contact of the ink with the bed and the moment when the blue colour appeared at the column outlet was measured.

## Supporting Information

File 1 Detailed analytical data of the prepared compounds and a collection of NMR spectra.
