# Supplementary material for: Efficient continuous-flow synthesis of novel 1,2,3-triazole-substituted β-aminocyclohexanecarboxylic acid derivatives with gram-scale production
Source: Beilstein J Org Chem. 2013 Jul 29;9:1508–16. doi: 10.3762/bjoc.9.172 (PMC3740622; doi:10.3762/bjoc.9.172)

Supporting Information

for

Efficient continuous-flow synthesis of novel 1,2,3-triazole-substituted β-aminocyclohexanecarboxylic acid derivatives with gram-scale production

Sándor B. Ötvös, Ádám Georgiádes, István M. Mándity, Lóránd Kiss and Ferenc Fülöp*

Address: Institute of Pharmaceutical Chemistry, University of Szeged, Eötvös u. 6, H-6720 Szeged, Hungary

E-mail: Ferenc Fülöp - fulop@pharm.u-szeged.hu

* Corresponding author

Detailed analytical data of the prepared compounds and a collection of NMR spectra.

Table of Contents

1. Analytical data S2

2. Collection of NMR spectra S6

1. Analytical data

1.1. Triazole 15 (Table 1, entry 1):

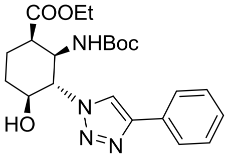

White solid; 144 mg (0.335 mmol) obtained; mp 175–178 °C; ^{1}H NMR (500.1 MHz, $CDCl_3$) δ_H: 1.29 (t, 3H, CH_3, J = 7.43 Hz), 1.43 (s, 9H, *t*-Bu), 1.75–1.89 (m, 1H, CH_2), 1.99–2.23 (m, 2H, CH_2), 2.26–2.39 (m, 1H, CH_2), 3.10–3.18 (m, 1H, CH), 3.85–3.95 (m, 1H, CH), 4.09–4.39 (m, 4H, 2CH and OCH_2), 5.72 (s, 1H, NH), 7.27–7.32 (m, 1H, Ar-H), 7.34–7.41 (m, 2H, Ar-H), 7.73–7.82 (m, 2H, Ar-H), 7.87 (s, 1H, Ar-H); ^{13}C NMR (125.0 MHz, $CDCl_3$) δ_C: 13.5, 25.0, 27.3, 27.9, 44.2, 54.9, 60.5, 65.3, 72.6, 79.5, 120.0, 125.2, 127.7, 128.3, 130.1, 147.0, 156.6, 173.0; elemental analysis calcd (%) for $C_{22}H_{30}N_4O_5$: C 61.38, H 7.02, N 13.01; found: C 61.43, H 7.00, N 13.00.

1.2. Triazole 16 (Table 1, entry 2):

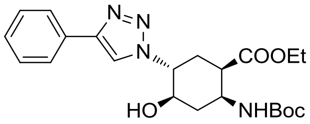

White solid; 132 mg (0.307 mmol) obtained; mp 204–206 °C; ^{1}H NMR (500.1 MHz, $CDCl_3$) δ_H: 1.31 (t, 3H, CH_3, J = 7.02 Hz), 1.46 (s, 9H, *t*-Bu), 1.91–2.04 (m, 1H, CH_2), 2.27–2.36 (m, 1H, CH_2), 2.37–2.46 (m, 1H, CH_2), 2.48–2.56 (m, 1H,CH_2), 3.10–3.22 (m, 1H, CH), 4.00–4.11 (m, 1H, CH), 4.17–4.32 (m, 3H, CH and OCH_2), 4.33–4.42 (m, 1H, CH), 5.48 (s, 1H, NH), 7.24–7.33 (m, 3H, Ar-H), 7.45–7.55 (m, 2H, Ar-H), 7.68 (s, 1H, Ar-H); ^{13}C NMR (125.0 MHz, $CDCl_3$) δ_C: 14.0, 27.9, 30.4, 36.0, 42.5, 47.3, 60.8, 62.8, 70.6, 79.4, 120.2, 125.0, 127.6, 128.2, 129.5, 146.2, 154.8, 172.4; elemental analysis calcd (%) for $C_{22}H_{30}N_4O_5$: C 61.38, H 7.02, N 13.01; found: C 61.36, H 7.02, N 13.03.

1.3. Triazole 17 (Table 1, entry 3):

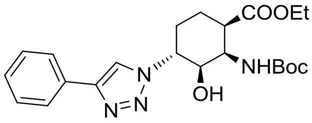

White solid; 190 mg (0.441 mmol) obtained; mp 176–178 °C; ^{1}H NMR (500.1 MHz, $CDCl_3$) δ_H: 1.28 (t, 3H, CH_3, J = 6.95 Hz), 1.44 (s, 9H, *t*-Bu), 1.71–1.86 (m, 1H, CH_2), 1.99–2.19 (m,

2H, CH_2), 2.23–2.38 (m, 1H, CH_2), 2.77–2.92 (m, 1H, CH), 4.10–4.27 (m, 3H, CH and OCH_2), 4.43–4.53 (m, 1H, CH), 4.57–4.65 (m, 1H, CH), 5.01 (s, 1H, NH), 7.31 (t, 1H, Ar-H, J = 7.22 Hz), 7.40 (t, 2H, Ar-H, J = 7.78 Hz), 7.81 (d, 2H, Ar-H, J = 7.56 Hz), 7.88 (s, 1H, Ar-H); ^{13}C NMR (125.0 MHz, $CDCl_3$) δ_C: 14.0, 21.3, 27.9, 28.6, 43.8, 52.5, 60.8, 60.9, 73.6, 80.8, 119.8, 125.3, 127.6, 128.3, 130.6, 146.7, 157.3, 171.5; elemental analysis calcd (%) for $C_{22}H_{30}N_4O_5$: C 61.38, H 7.02, N 13.01; found: C 61.44, H 7.00, N 12.98.

1.4. Triazole 18 (Table 1, entry 4):

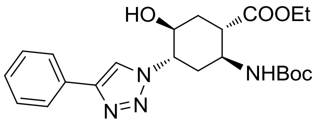

White solid; 220 mg (0.511 mmol) obtained; mp 173–176 °C; ^{1}H NMR (500.1 MHz, $CDCl_3$) δ_H: 1.32 (t, 3H, CH_3, J = 7.05 Hz), 1.50 (s, 9H, *t*-Bu), 1.90–2.07 (m, 1H, CH_2), 2.27–2.38 (m, 1H, CH_2), 2.40–2.56 (m, 2H, CH_2), 3.00–3.10 (m, 1H, CH), 4.15–4.35 (m, 3H, CH and OCH_2), 4.40–4.51 (m, 1H, CH), 4.57–4.72 (m, 1H, CH), 5.25 (s, 1H, NH), 730–7.43 (m, 3H, Ar-H), 7.72 (d, 2H, Ar-H, J = 4.10 Hz), 7.84 (s, 1H, Ar-H); ^{13}C NMR (125.0 MHz, $CDCl_3$) δ_C: 13.9, 28.1, 30.3, 32.1, 43.4, 46.6, 60.8, 61.6, 68.8, 79.8, 119.0, 125.2, 127.8, 128.4, 129.6, 147.0, 155.8, 172,2; elemental analysis calcd (%) for $C_{22}H_{30}N_4O_5$: C 61.38, H 7.02, N 13.01; found: C 61.39, H 7.03, N 12.97.

1.5. Triazole 19 (Table 1, entry 5):

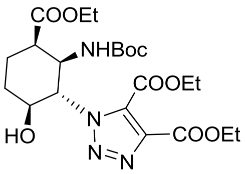

Pale yellow oil; 207 mg (0.415 mmol) obtained; ^{1}H NMR (500.1 MHz, $CDCl_3$) δ_H: 1.34 (t, 3H, CH_3, J = 7.20 Hz), 1.41 (t, 6H, $2CH_3$, J = 7.10 Hz), 1.45 (s, 9H, *t*-Bu), 1.70–1.91 (m, 1H, CH_2), 2.06–2.19 (m, 1H, CH_2), 2.23–2.36 (m, 1H, CH_2), 2.37–2.49 (m, 1H,CH_2), 3.07–3.17 (m, 1H, CH), 3.78–3.93 (m, 1H, CH), 4.16–4.38 (m, 3H, CH and OCH_2), 4.38–4.51 (m, 4H, $2OCH_2$), 4.70–4.81 (m, 1H, CH), 5.78 (s, 1H, NH); ^{13}C NMR (125.0 MHz, $CDCl_3$) δ_C: 13.4, 13.7, 25.3, 26.8, 27.8, 43.9, 54.5, 60.9, 61.2, 62.4, 64.1, 72.8, 79.8, 131.3, 138.9, 156.4, 158.8, 159.8, 172.4; elemental analysis calcd (%) for $C_{22}H_{34}N_4O_9$: C 53.00, H 6.87, N 11.24; found: C 53.05, H 6.85, N 11.21.

1.6. Triazole 20 (Table 1, entry 6):

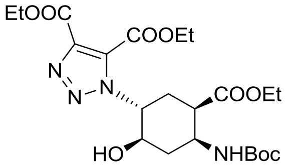

Pale yellow solid; 208 mg (0.417 mmol) obtained; mp 63–66 °C; ^{1}H NMR (500.1 MHz, $CDCl_3$) δ_H: 1.37 (t, 3H, CH_3, J = 7.44 Hz), 1.43 (t, 6H, $2CH_3$, J = 7.40 Hz), 1.48 (s, 9H, *t*-Bu), 1.92–2.09 (m, 1H, CH_2), 2.26–2.35 (m, 1H, CH_2), 2.39–2.51 (m, 1H, CH_2), 2.55–2.65 (m, 1H,CH_2), 3.11–3.22 (m, 1H, CH), 3.97–4.09 (m, 1H, CH), 4.24–4.37 (m, 3H, CH and OCH_2), 4.40–4.52 (m, 4H, $2OCH_2$), 4.84–4.97 (m, 1H, CH), 5.52 (s, 1H, NH); ^{13}C NMR (125.0 MHz, $CDCl_3$) δ_C: 13.4, 13.7, 27.9, 30.4, 36.4, 42.6, 47.3, 60.8, 61.4, 61.6, 62.5, 70.4, 79.3, 131.4, 139.1, 154.7, 158.3, 159.7, 172.1; elemental analysis calcd (%) for $C_{22}H_{34}N_4O_9$: C 53.00, H 6.87, N 11.24; found: C 52.97, H 6.86, N 11.26.

1.7. Triazole 21 (Table 1, entry 7):

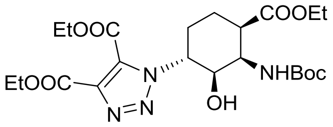

White solid; 205 mg (0.411 mmol) obtained; mp 157–159 °C; ^{1}H NMR (500.1 MHz, $CDCl_3$) δ_H: 1.26 (t, 3H, CH_3, J = 7.24 Hz), 1.37 (t, 6H, $2CH_3$, J = 8.10 Hz), 1.44 (s, 9H, *t*-Bu), 1.75–1.88 (m, 1H, CH_2), 2.02–2.14 (m, 1H, CH_2), 2.21–2.32 (m, 1H, CH_2), 2.37–2.50 (m, 1H,CH_2), 2.81–2.93 (m, 1H, CH), 3.92–4.00 (m, 1H, CH), 4.11–4.27 (m, 2H, OCH_2), 4.34–4.46 (m, 4H, $2OCH_2$), 4.48–4.55 (m, 1H, CH), 4.88–4.97 (m, 1H, CH), 5.01 (s, 1H, NH); ^{13}C NMR (125.0 MHz, $CDCl_3$) δ_C: 13.4, 13.7, 21.4, 27.2, 27.7, 43.5, 52.7, 59.5, 60.9, 61.2, 62.1, 74.7, 80.8, 131.7, 138.9, 157.9, 159.1, 159.9, 171.4; elemental analysis calcd (%) for $C_{22}H_{34}N_4O_9$: C 53.00, H 6.87, N 11.24; found: C 53.04, H 6.86, N 11.22.

1.8. Triazole 22 (Table 1, entry 8):

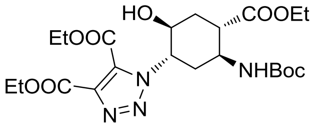

Pale yellow solid; 2.24 g (4.49 mmol) obtained; mp 145–148 °C; ^{1}H NMR (500.1 MHz, $CDCl_3$) δ_H: 1.33 (t, 3H, CH_3, J = 7.47 Hz), 1.44 (t, 6H, $2CH_3$, J = 7.40 Hz), 1.51 (s, 9H, *t*-Bu), 1.81–1.94 (m, 1H, CH_2), 2.28–2.34 (m, 1H, CH_2), 2.41–2.60 (m, 2H, CH_2), 3.08–3.17 (m, 1H, CH), 4.20–4.32 (m, 2H, OCH_2), 4.40–4.53 (m, 6H, 2CH and $2OCH_2$), 4.80–4.91 (m, 2H, CH

and NH); ^{13}C NMR (125.0 MHz, $CDCl_3$) δ_C: 13.4, 13.8, 28.0, 30.0, 32.2, 43.3, 46.7, 60.9, 61.4, 61.5, 62.6, 68.3, 79.7, 130.6, 139.4, 154.7, 158.6, 159.8, 171.6; elemental analysis calcd (%) for $C_{22}H_{34}N_4O_9$: C 53.00, H 6.87, N 11.24; found: C 52.98, H 6.89, N 11.27.

1.9. Triazole 23 (Table 1, entry 9):

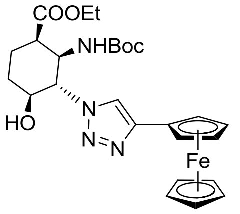

Golden brown solid; 220 mg (0.409 mmol) obtained; mp 195–198 °C; ^1H NMR (500.1 MHz, $CDCl_3$) δ_H: 1.29 (t, 3H, CH_3, J = 7.04 Hz), 1.44 (s, 9H, *t*-Bu), 1.75–1.89 (m, 1H, CH_2), 2.00–2.25 (m, 2H, CH_2), 2.27–2.41 (m, 1H, CH_2), 3.06–3.19 (m, 1H, CH), 3.84–3.95 (m, 1H, CH), 4.07 (s, 5H, Ar-H), 4.10–4.26 (m, 3H, CH and OCH_2), 4.26–4.36 (m, 3H, Ar-H and CH), 4.71 (s, 2H, Ar-H), 5.67 (s, 1H, NH), 7.56 (s, 1H, Ar-H); ^{13}C NMR (125.0 MHz, $CDCl_3$) δ_C: 13.8, 25.2, 27.2, 27.9, 43.9, 54.4, 60.5, 60.9, 64.6, 68.4, 69.3, 72.4, 75.1, 79.8, 119.2, 145.8, 156.3, 173.2; elemental analysis calcd (%) for $C_{26}H_{34}FeN_4O_5$: C 58.00, H 6.36, N 10.41; found: C 58.05, H 6.35, N 10.38.

1.10. Triazole 24 (Table 1, entry 10):

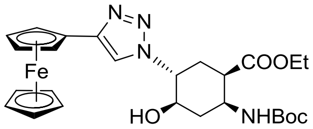

Golden brown solid; 217 mg (0.403 mmol) obtained; mp 193–195 °C; ^1H NMR (500.1 MHz, $CDCl_3$) δ_H: 1.37 (t, 3H, CH_3, J = 7.25 Hz), 1.50 (s, 9H, *t*-Bu), 1.94–2.06 (m, 1H, CH_2), 2.23–2.38 (m, 2H, CH_2), 2.57–2.66 (m, 1H, CH_2), 3.17–3.23 (m, 1H, CH), 3.99–4.11 (m, 6H, 5Ar-H and CH), 4.21–4.35 (m, 5H, 4Ar-H and OCH_2), 4.36–4.46 (m, 1H, OCH_2), 4.57–4.65 (m, 1H, CH), 4.66–4.75 (m, 1H, CH), 5.45 (s, 1H, NH), 7.48 (s, 1H, Ar-H); ^{13}C NMR (125.0 MHz, $CDCl_3$) δ_C: 13.9, 28.1, 30.3, 35.9, 42.7, 47.5, 60.8, 62.5, 66.4, 68.5, 69.5, 70.3, 75.0, 79.3, 119.3, 145.5, 154.6, 172.6; elemental analysis calcd (%) for $C_{26}H_{34}FeN_4O_5$: C 58.00, H 6.36, N 10.41; found: C 58.05, H 6.37, N 10.36.

1.11. Triazole 25 (Table 1, entry 11):

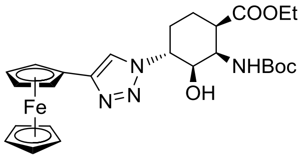

Golden brown solid; 217 mg (0.403 mmol) obtained; mp 201–203 °C; ^{1}H NMR (500.1 MHz, $CDCl_3$) δ_H: 1.32 (t, 3H, CH_3, J = 7.29 Hz), 1.49 (s, 9H, *t*-Bu), 1.77–1.89 (m, 1H, CH_2), 2.03–2.21 (m, 2H, CH_2), 2.30–2.41 (m, 1H, CH_2), 2.85–2.94 (m, 1H, CH), 4.12 (s, 5H, Ar-H), 4.15–4.30 (m, 3H, OCH_2 and CH), 4.33 (s, 2H, Ar-H), 4.41–4.51 (m, 1H, CH), 4.60–4.68 (m, 1H, CH), 4.76 (s, 2H, Ar-H), 5.00 (s, 1H, NH), 7.59 (s, 1H, Ar-H); ^{13}C NMR (125.0 MHz, $CDCl_3$) δ_C: 13.6, 21.2, 27.8, 28.5, 43.8, 52.4, 60.5, 60.9, 66.5, 68.1, 69.2, 73.2, 75.3, 80.5, 119.1, 145.7, 157.2, 171.6; elemental analysis calcd (%) for $C_{26}H_{34}FeN_4O_5$: C 58.00, H 6.36, N 10.41; found: C 57.96, H 6.38, N 10.43

1.12. Triazole 26 (Table 1, entry 12):

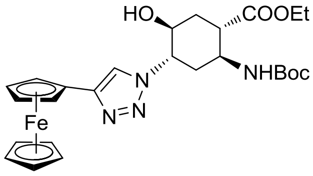

Golden brown solid; 197 mg (0.367 mmol) obtained; mp 87–90 °C; ^{1}H NMR (500.1 MHz, $CDCl_3$) δ_H: 1.33 (t, 3H, CH_3, J = 7.17 Hz), 1.52 (s, 9H, *t*-Bu), 1.87–2.01 (m, 1H, CH_2), 2.32–2.45 (m, 2H, CH_2), 2.46–2.56 (m, 1H, CH_2), 3.01–3.09 (m, 1H, CH), 4.11 (s, 5H, Ar-H), 4.15–4.30 (m, 3H, OCH_2, CH), 4.34 (s, 2H, Ar-H), 4.42–4.56 (m, 2H, 2CH), 4.73, (s, 2H, Ar-H), 4.90 (s, 1H, NH), 7.60 (s, 1H, Ar-H); ^{13}C NMR (125.0 MHz, $CDCl_3$) δ_C: 13.8, 28.0, 29.9, 31.9, 43.3, 46.4, 60.8, 61.3, 66.4, 68.4, 68.5, 69.4, 75.0, 79.5, 117.7, 146.2, 154.8, 172.2; elemental analysis calcd (%) for $C_{26}H_{34}FeN_4O_5$: C 58.00, H 6.36, N 10.41; found: C 58.02, H 6.37, N 10.38

2. Collection of NMR spectra

2.1. Triazole 15 ^{1}H and ^{13}C NMR in $CDCl_3$:

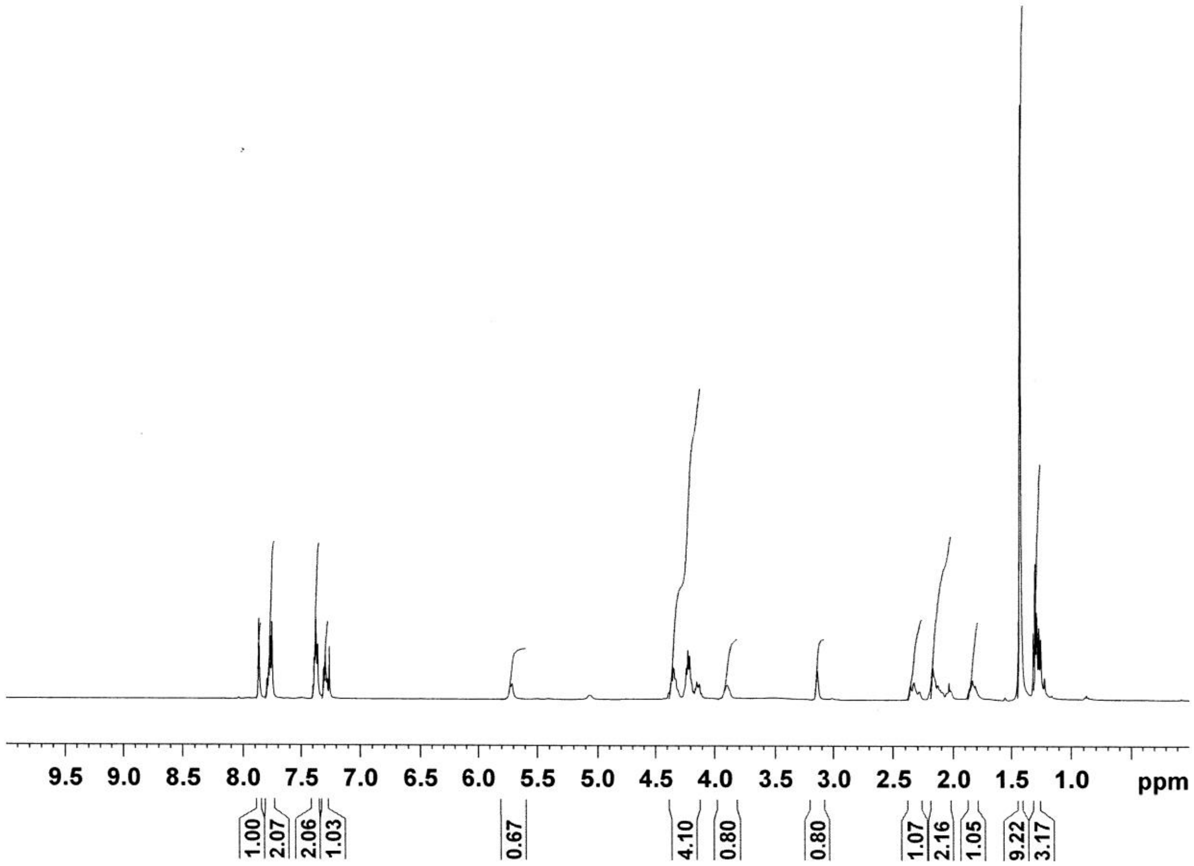

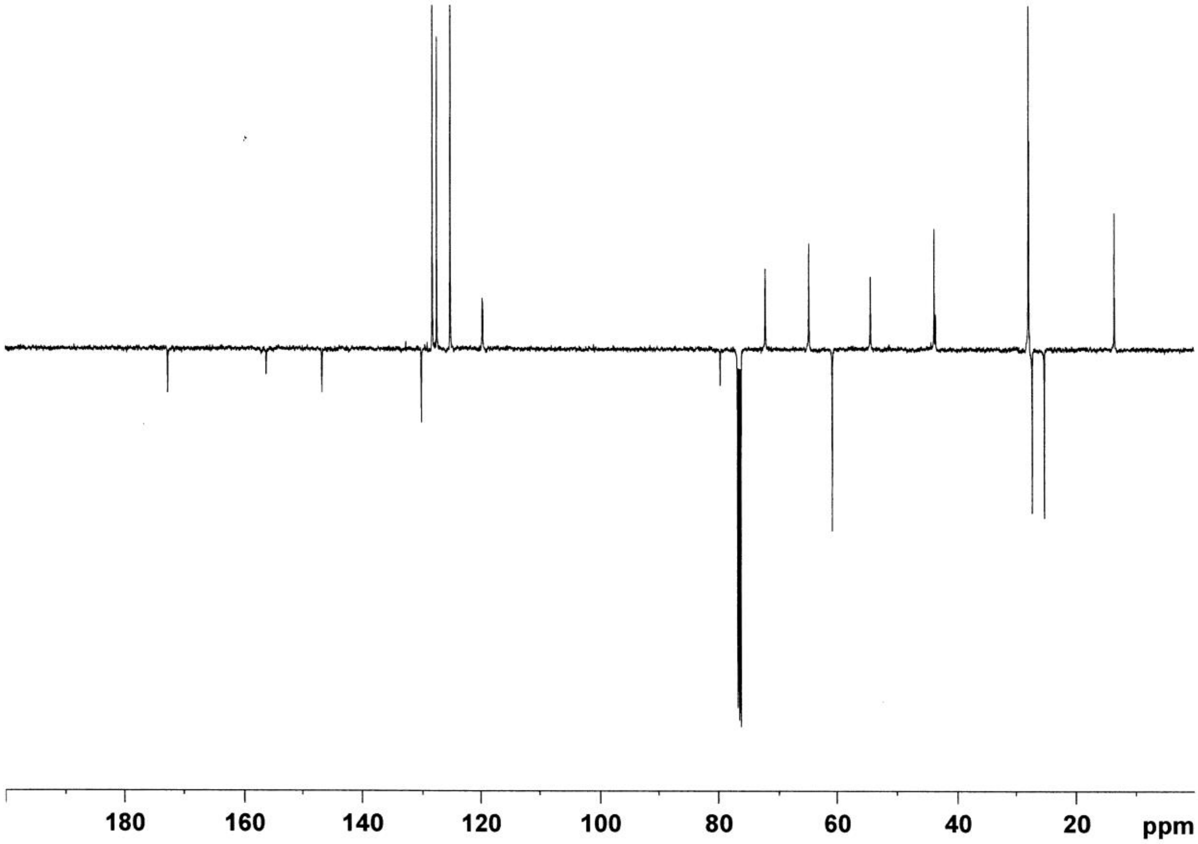

2.2. Triazole 16 ^{1}H and ^{13}C NMR in $CDCl_3$:

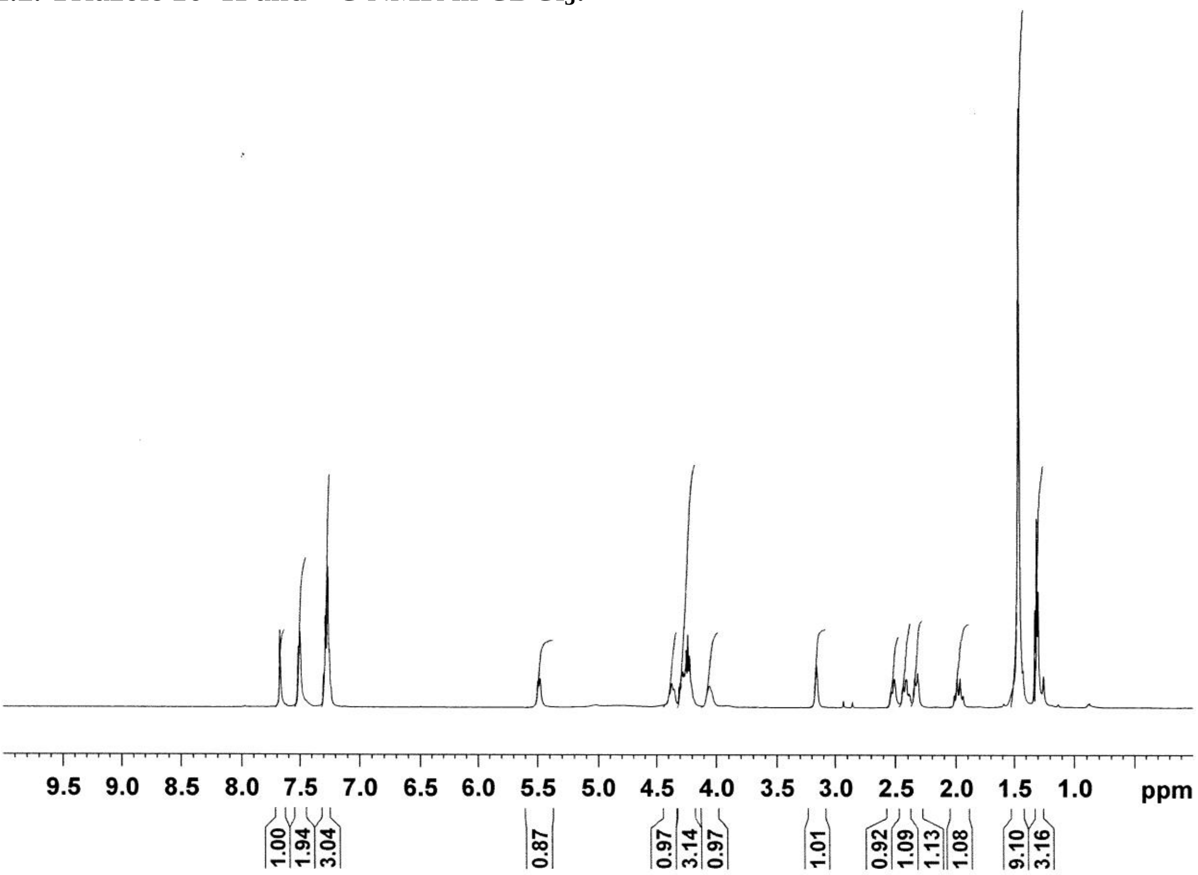

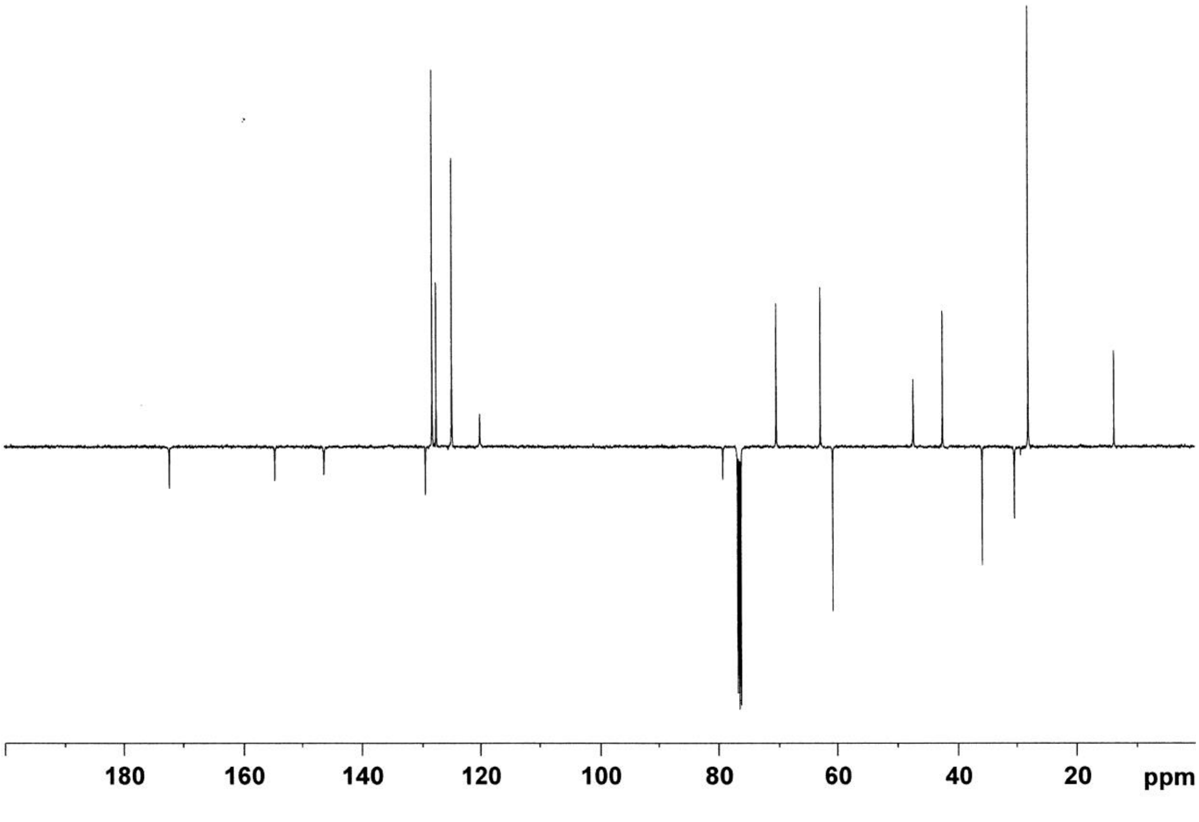

2.3. Triazole 17 ^{1}H and ^{13}C NMR in $CDCl_3$:

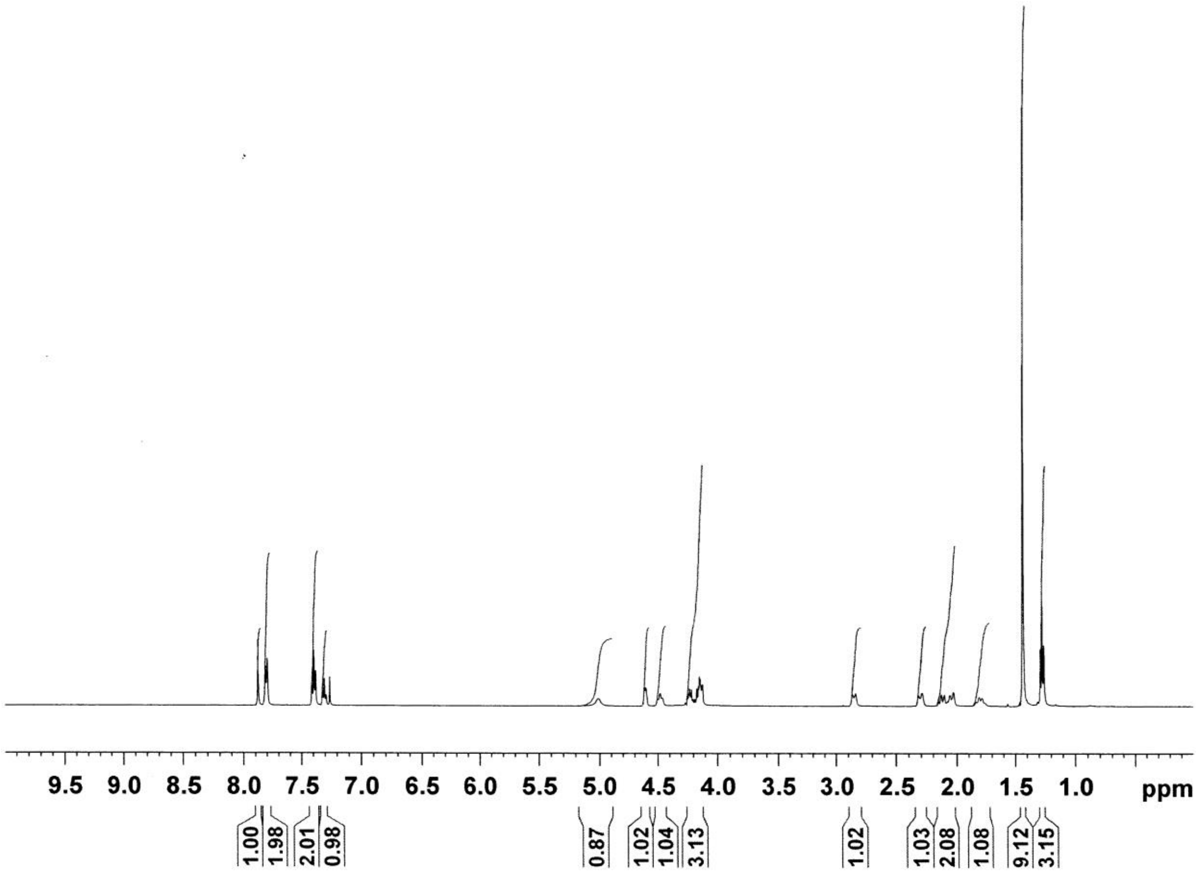

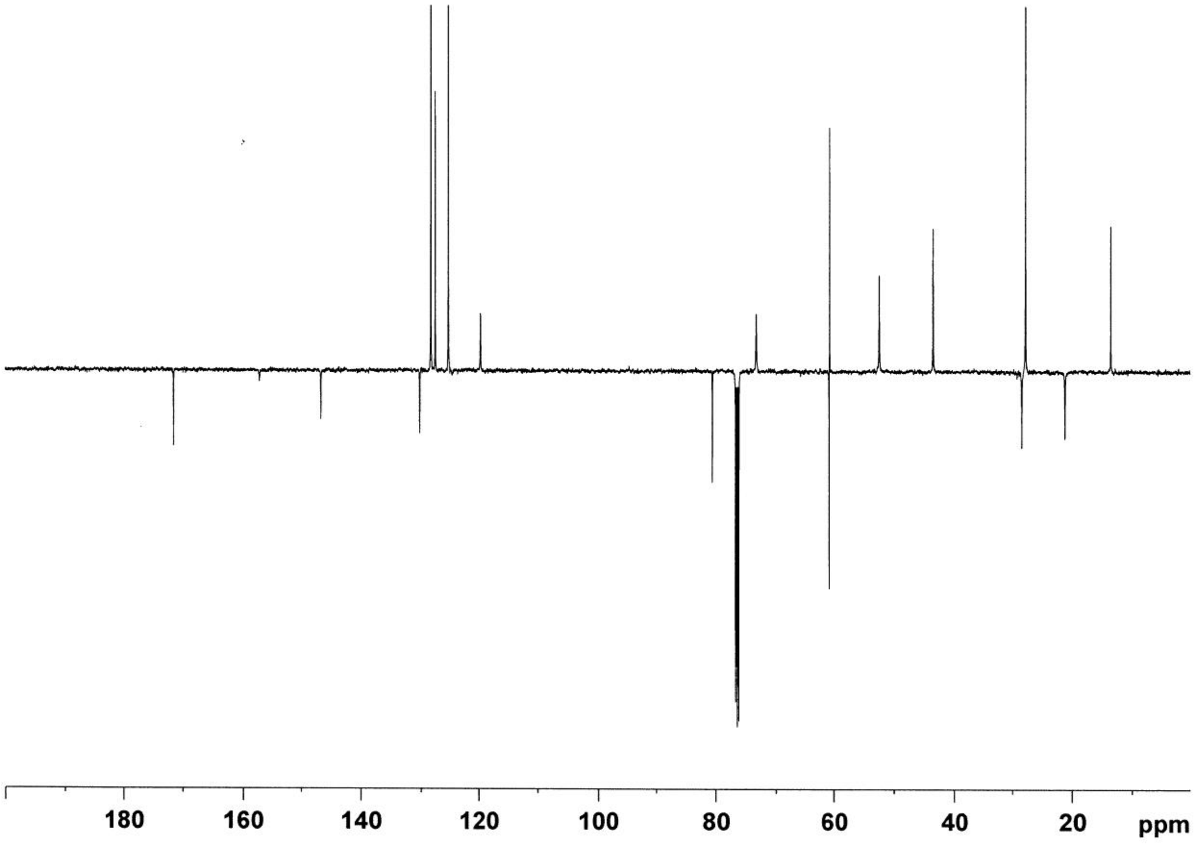

2.4. Triazole 18 ^{1}H and ^{13}C NMR in $CDCl_3$:

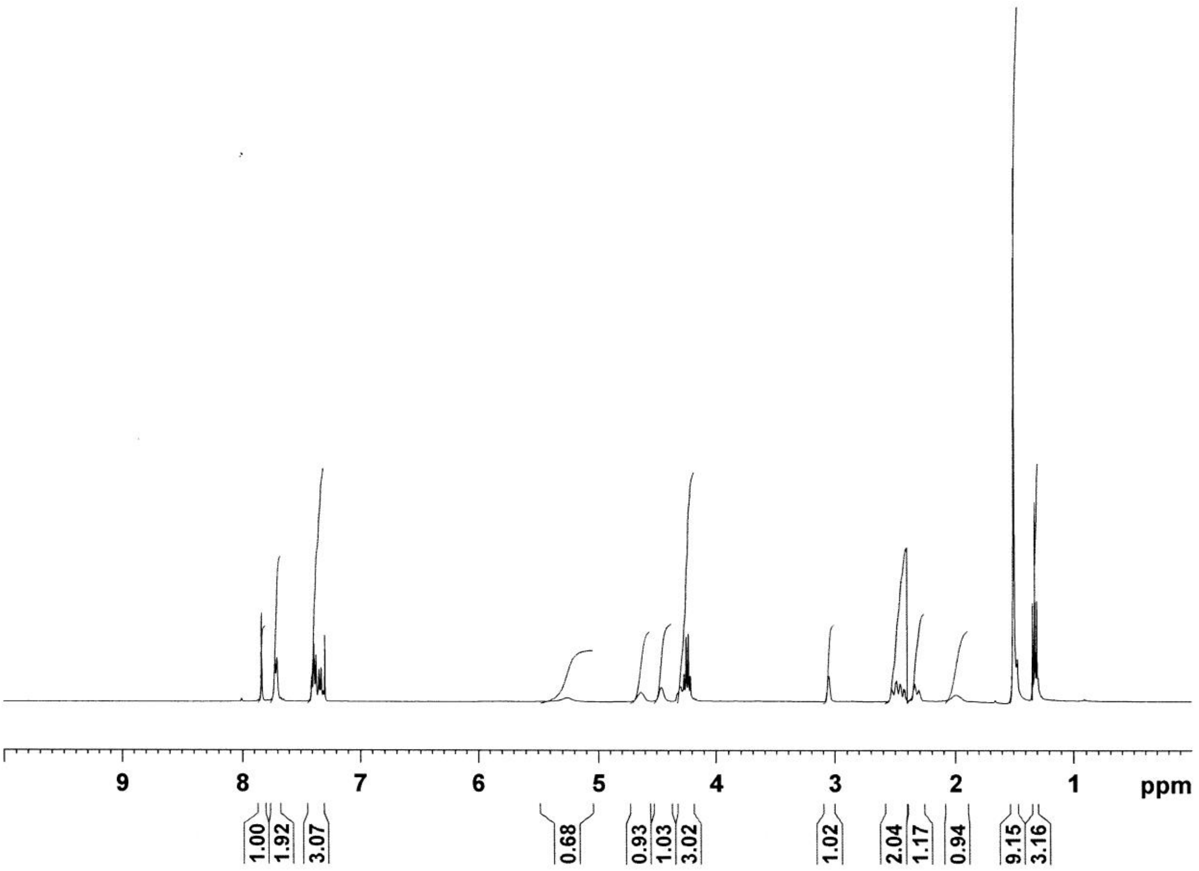

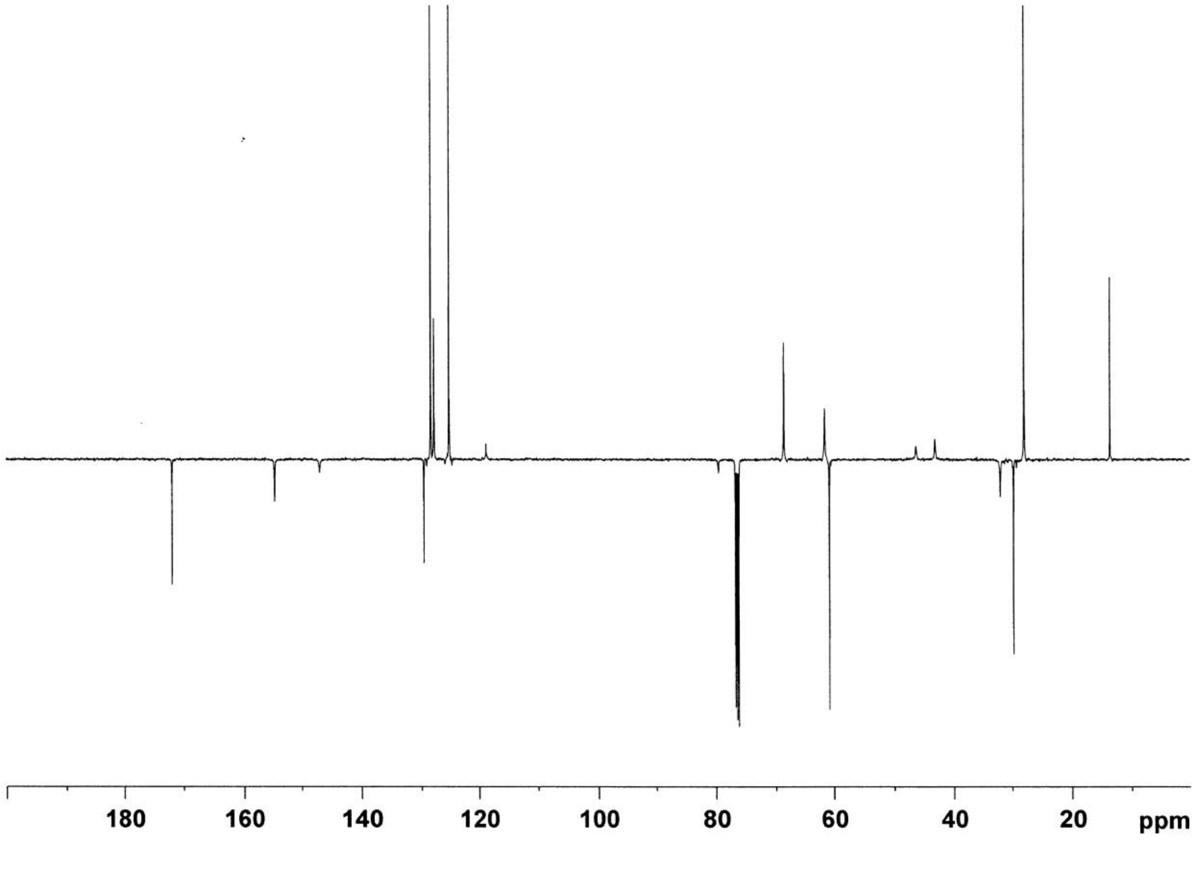

2.5. Triazole 19 ^{1}H and ^{13}C NMR in $CDCl_3$:

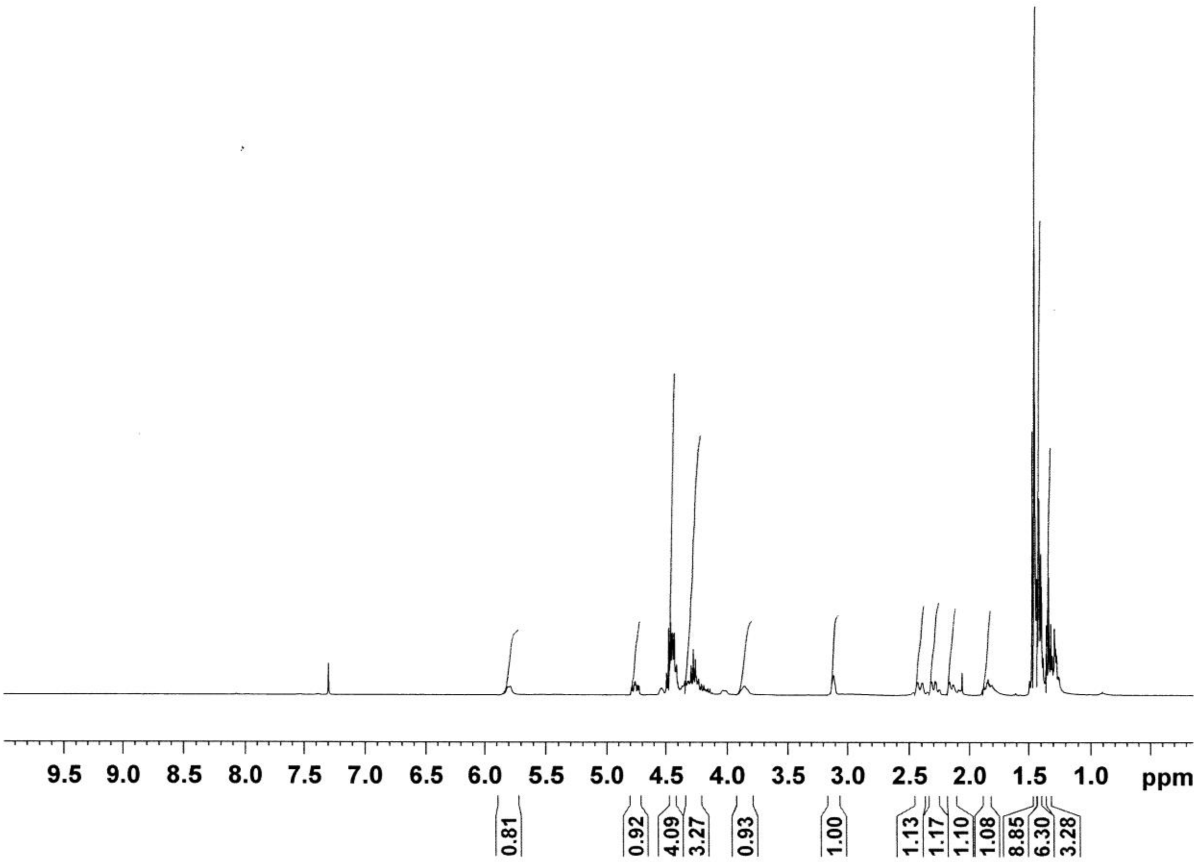

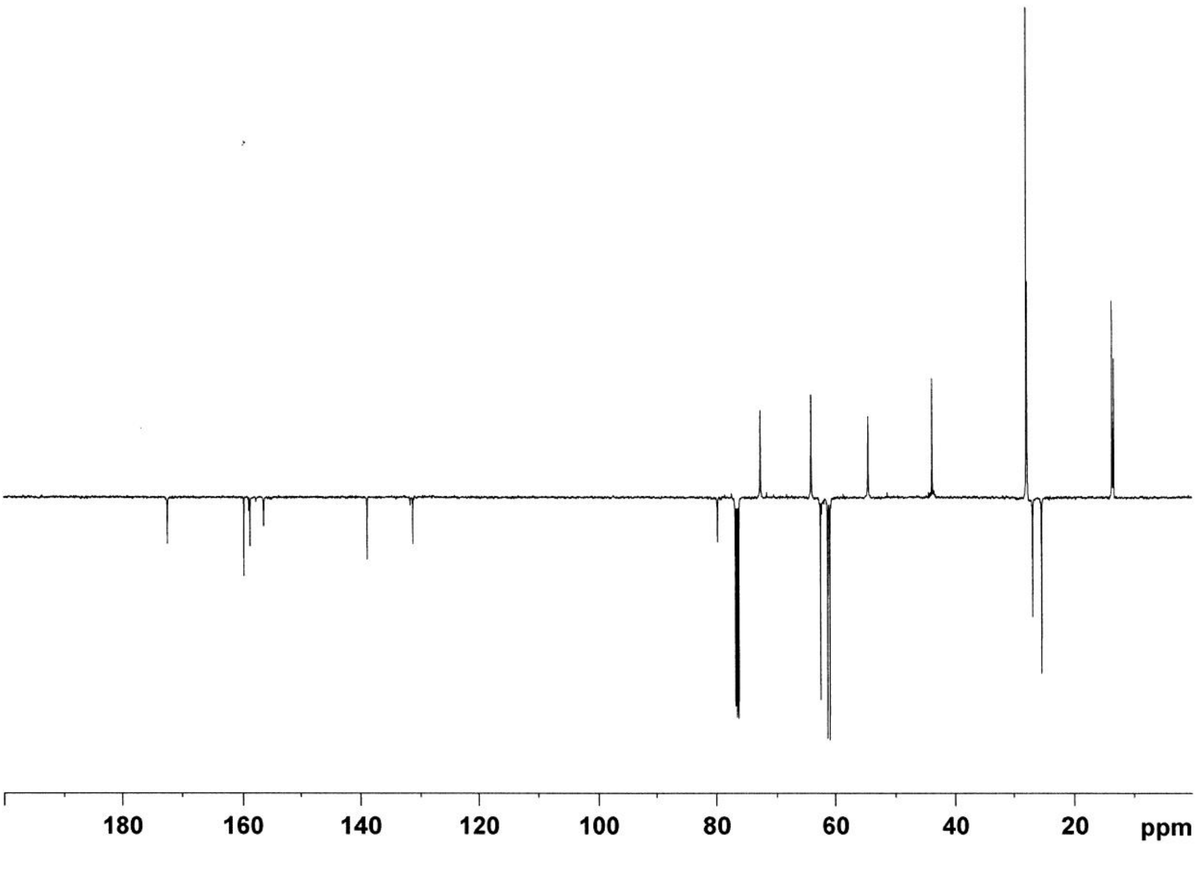

2.6. Triazole 20 ^{1}H and ^{13}C NMR in $CDCl_3$:

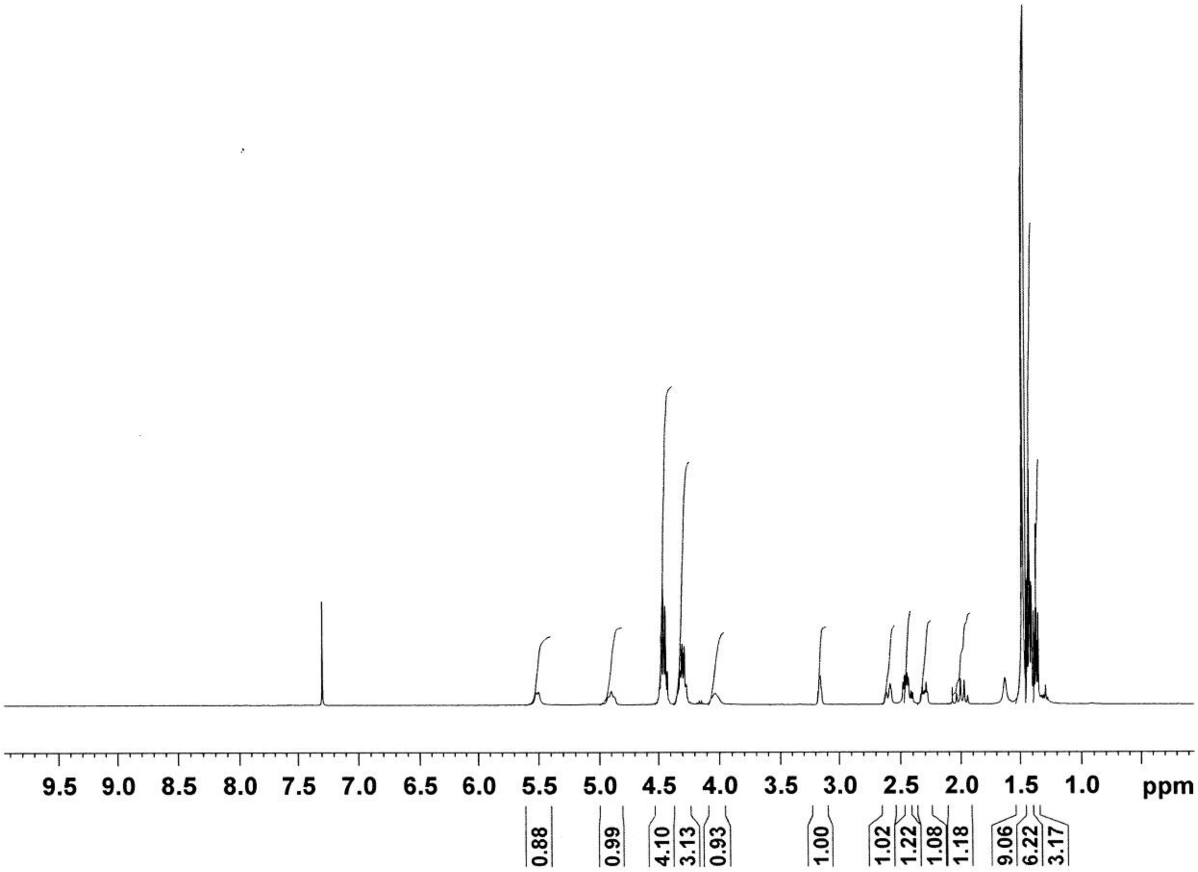

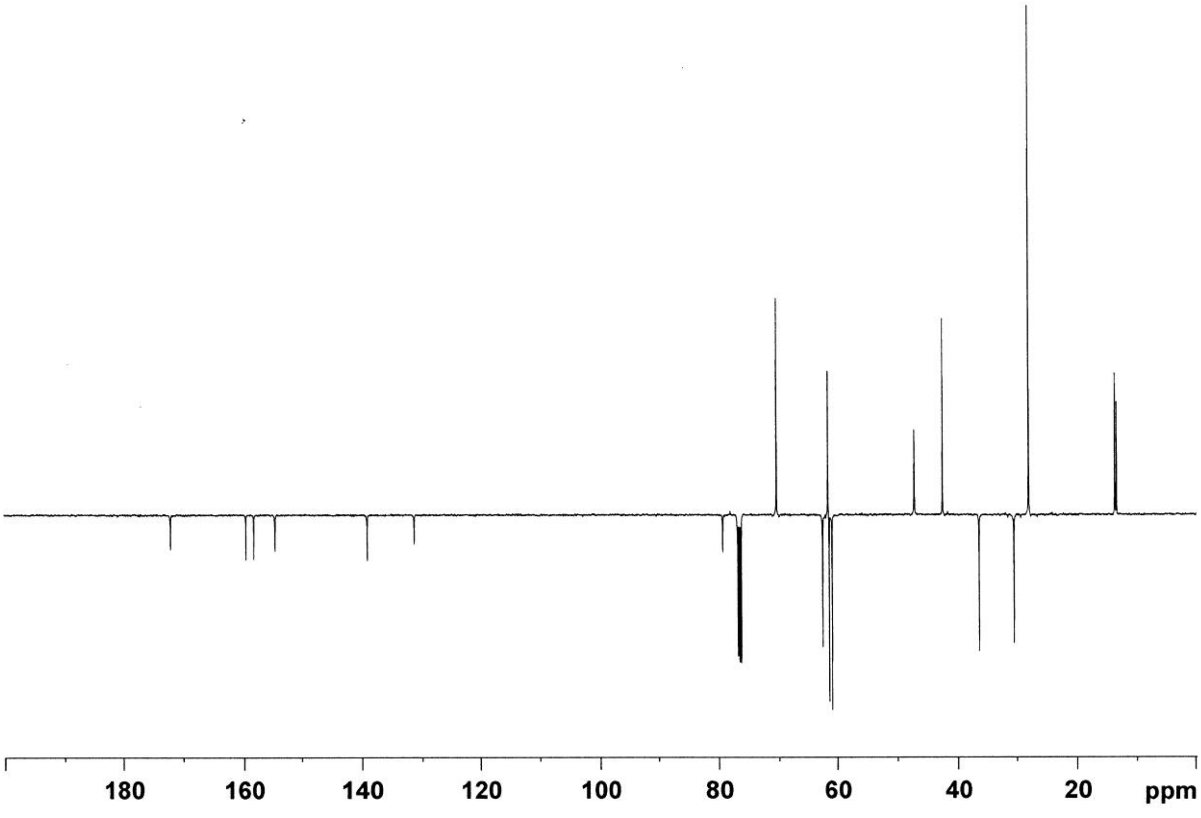

2.7. Triazole 21 ^{1}H and ^{13}C NMR in $CDCl_3$:

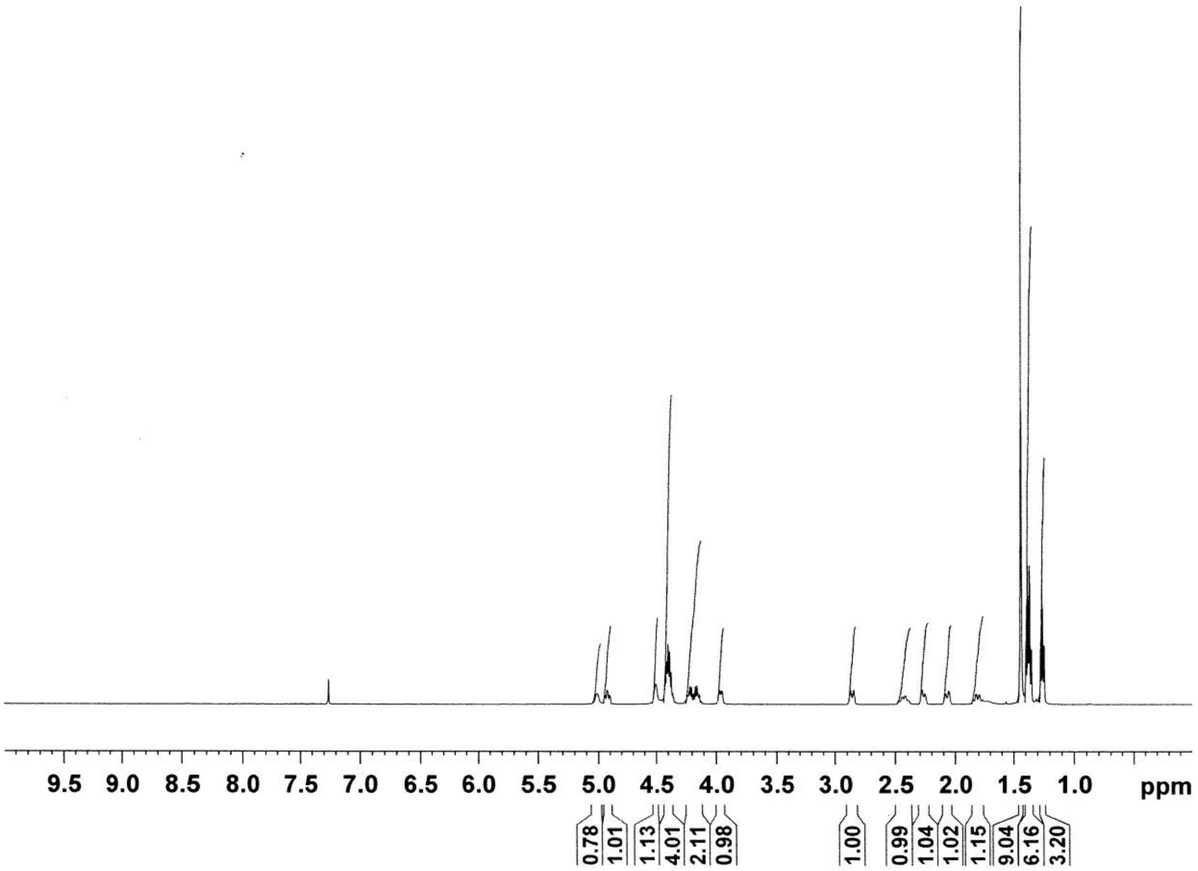

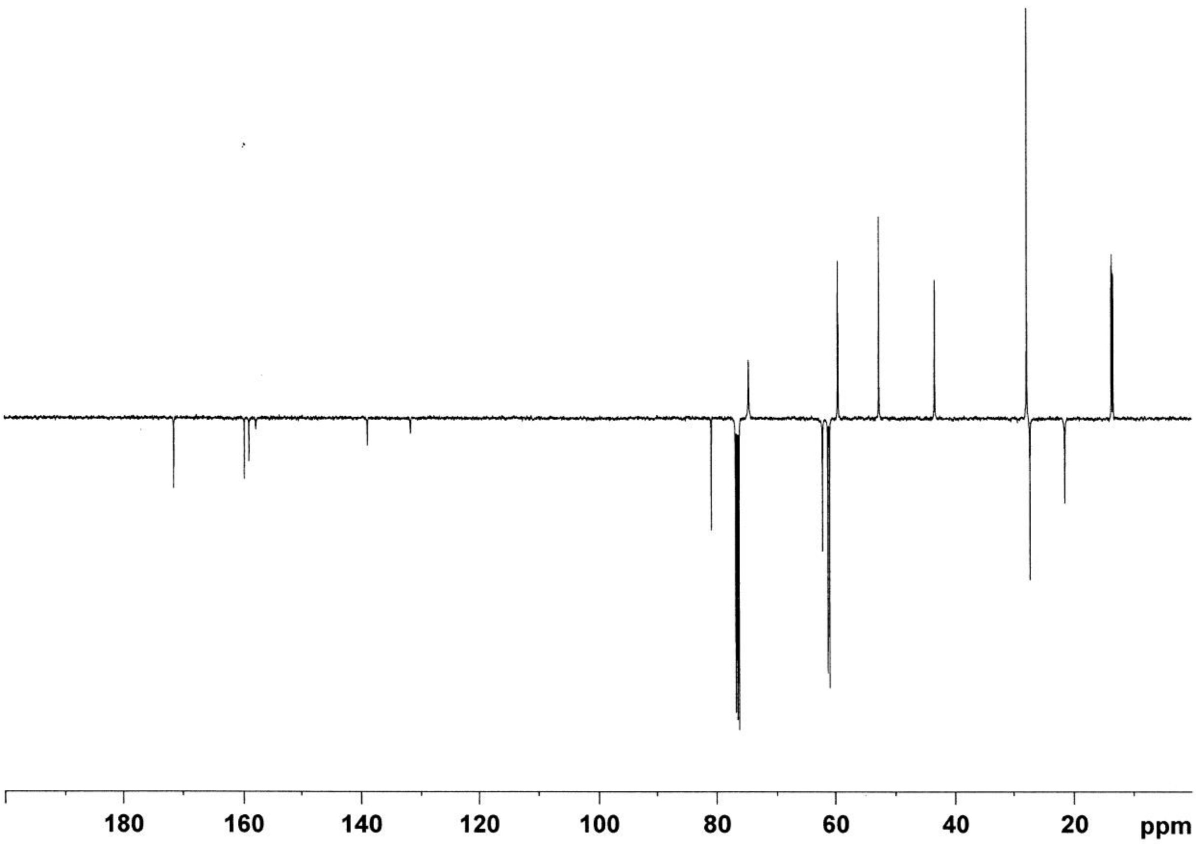

2.8. Triazole 22 ^{1}H and ^{13}C NMR in $CDCl_3$:

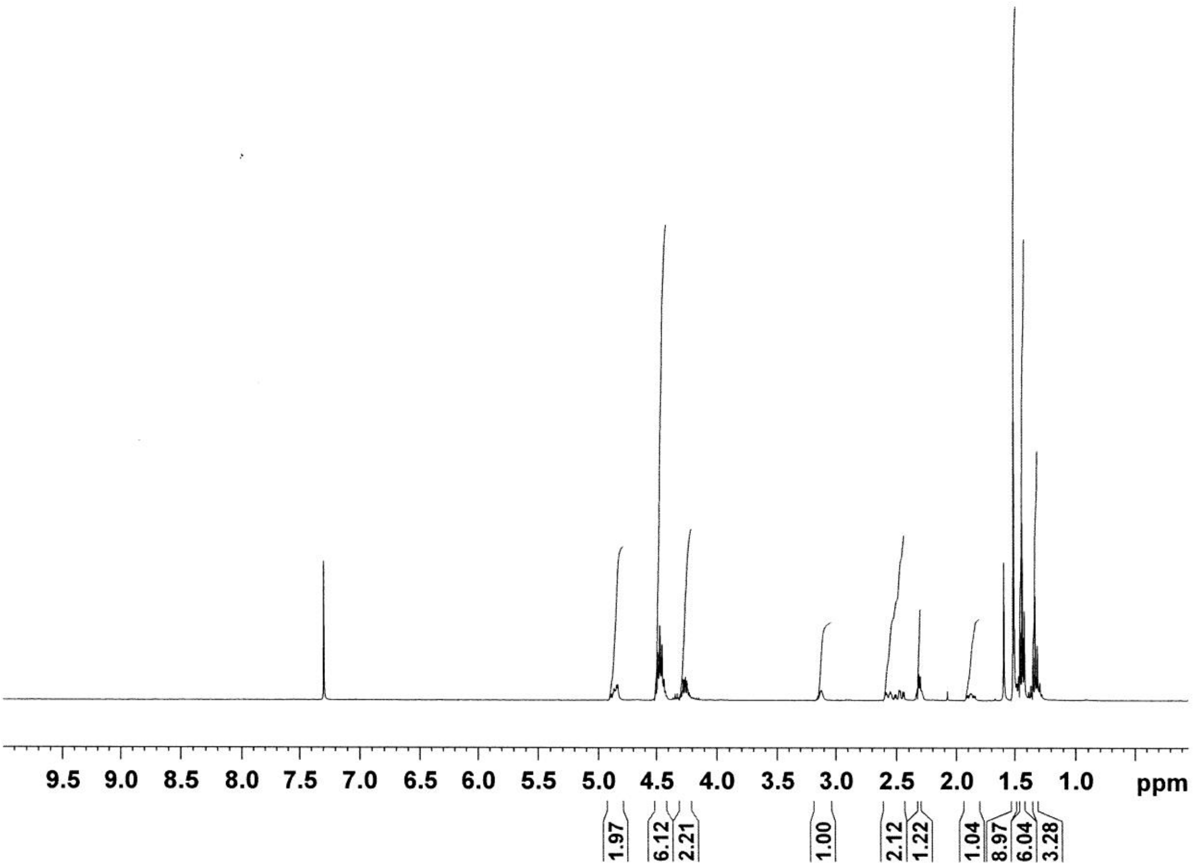

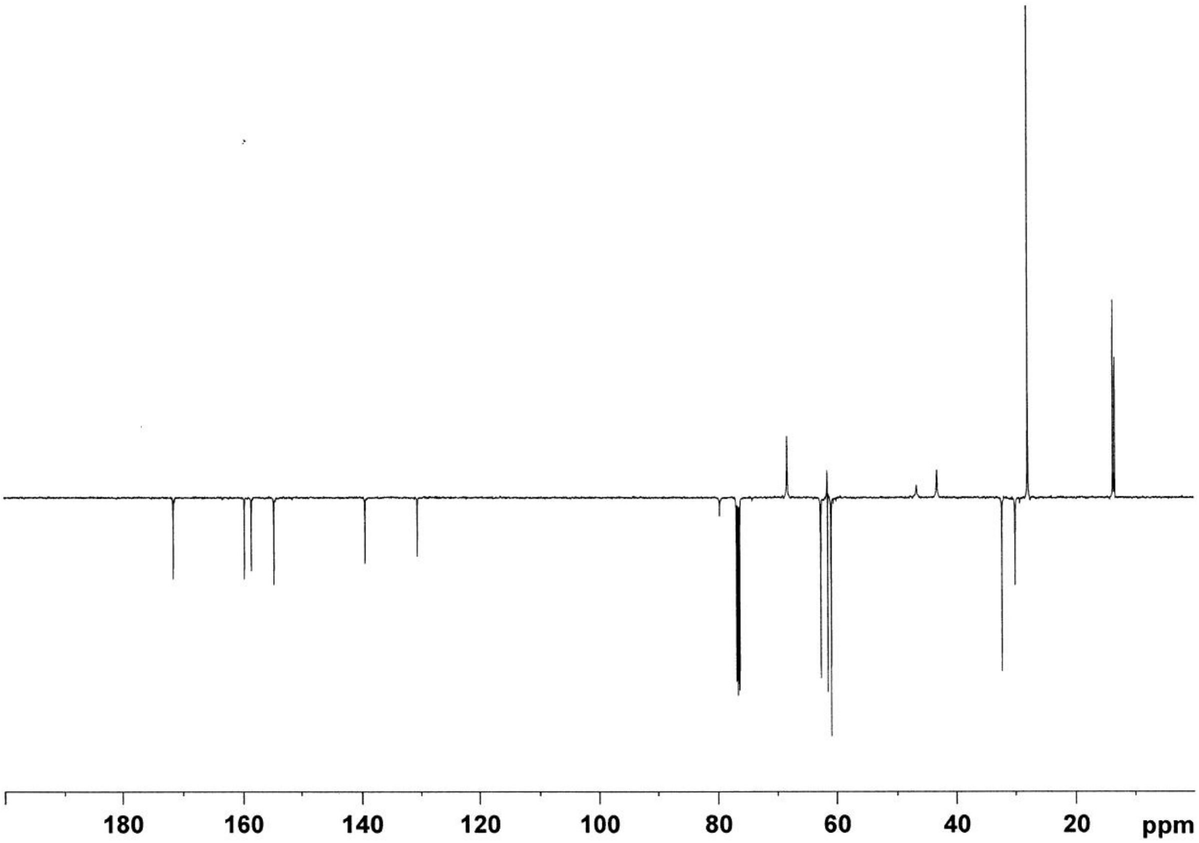

2.9. Triazole 23 ^{1}H and ^{13}C NMR in $CDCl_3$:

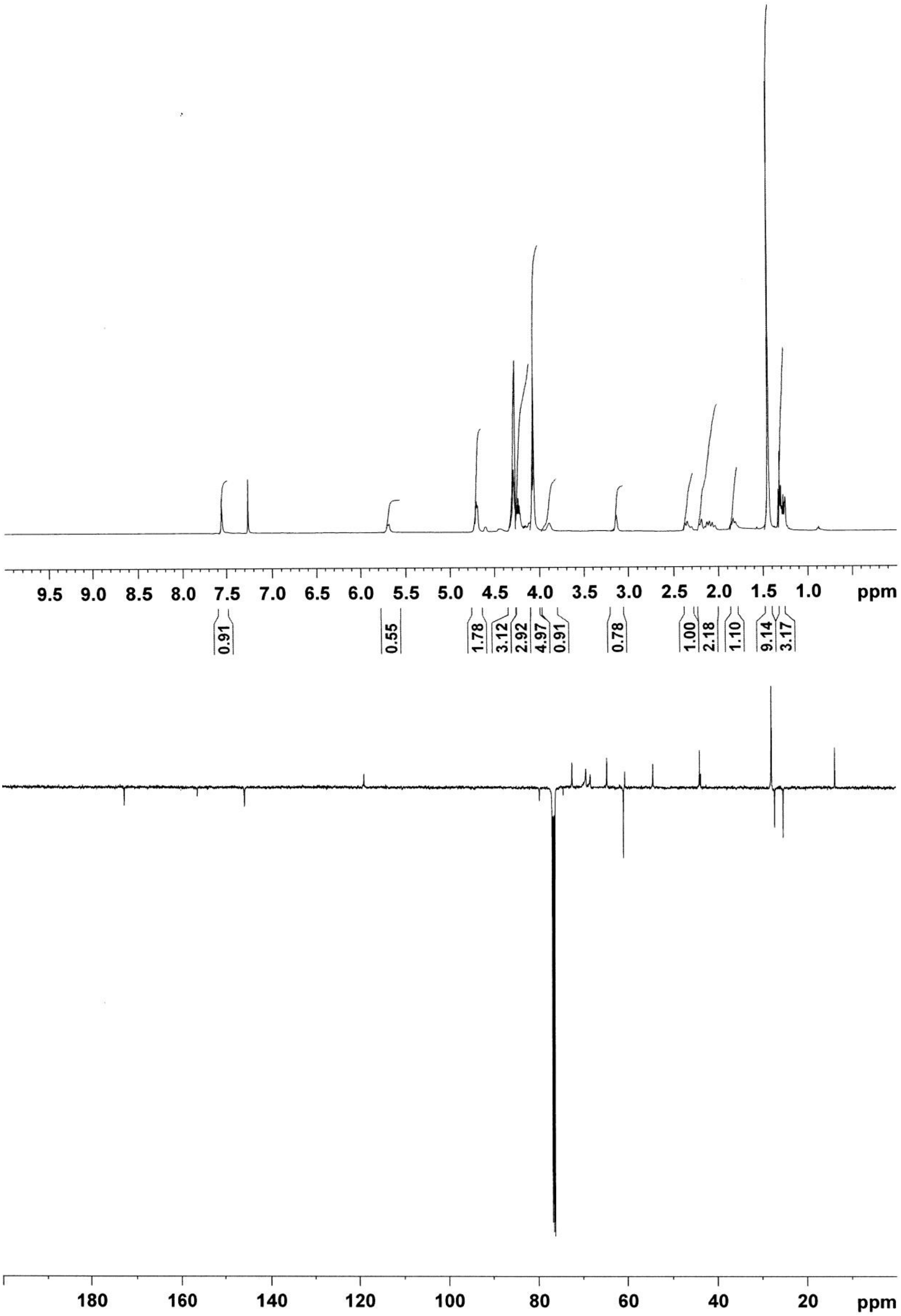

2.10. Triazole 24 ^{1}H and ^{13}C NMR in $CDCl_3$:

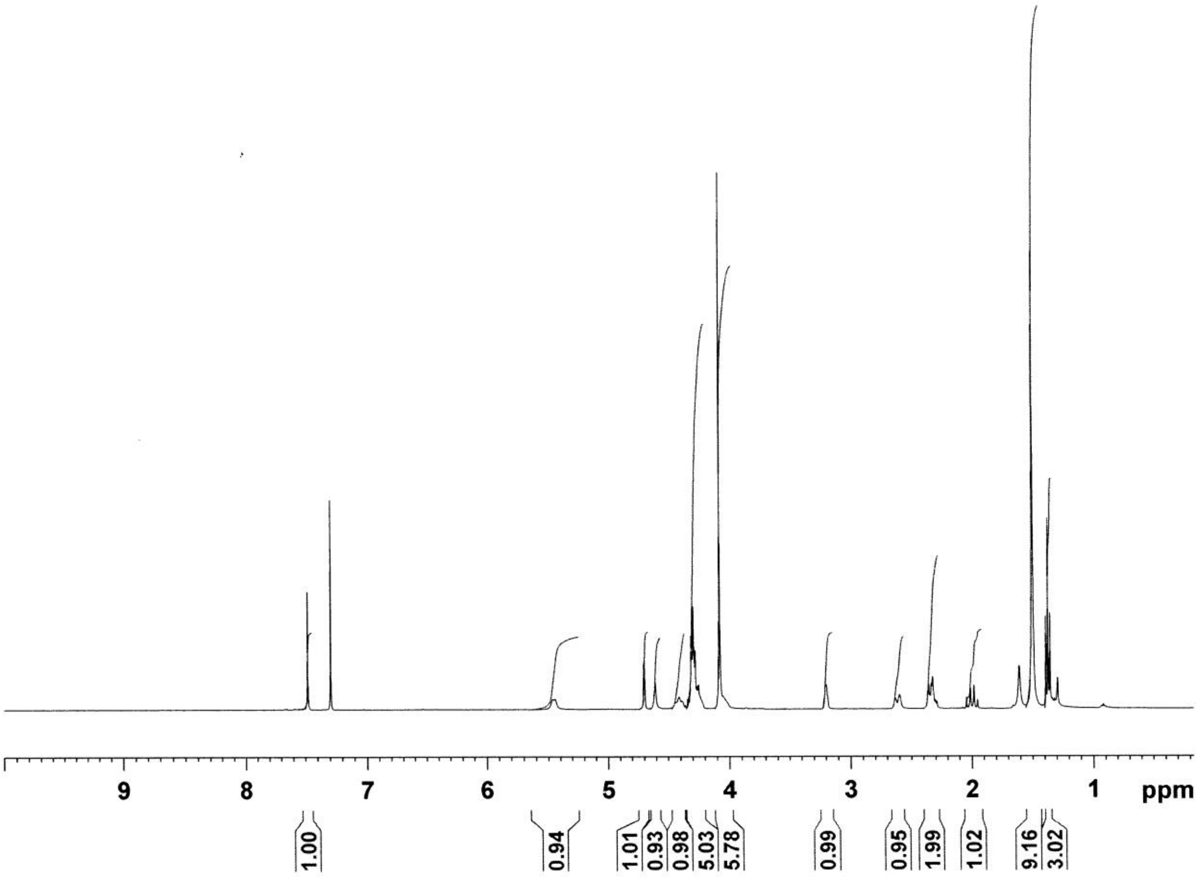

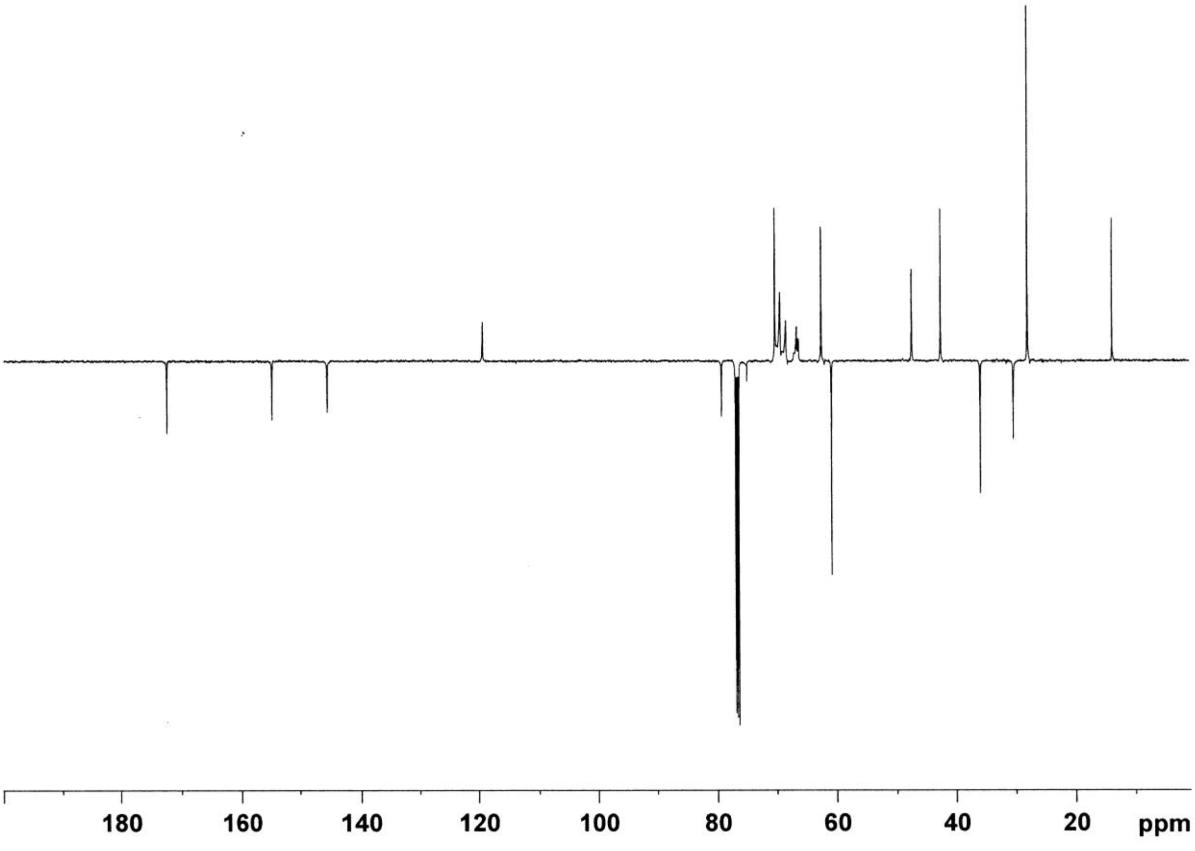

2.11. Triazole 25 ^{1}H and ^{13}C NMR in $CDCl_3$:

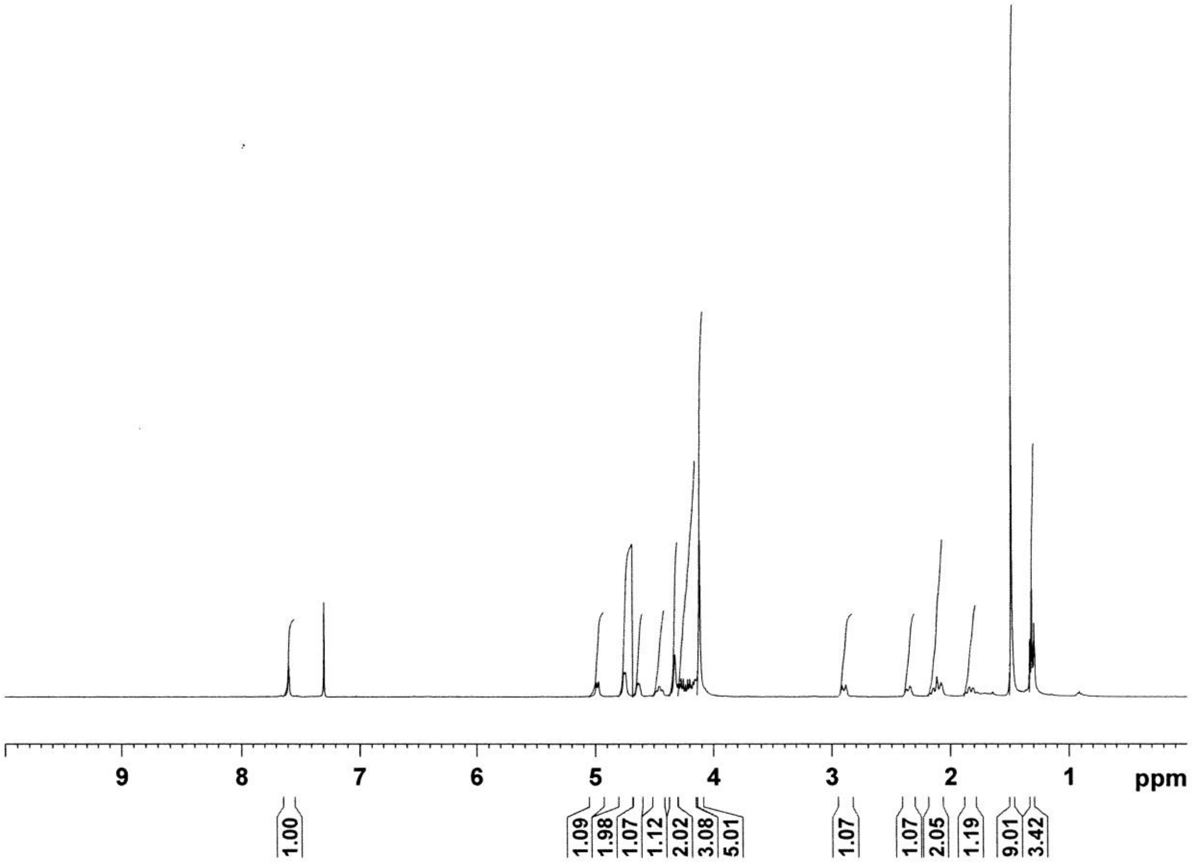

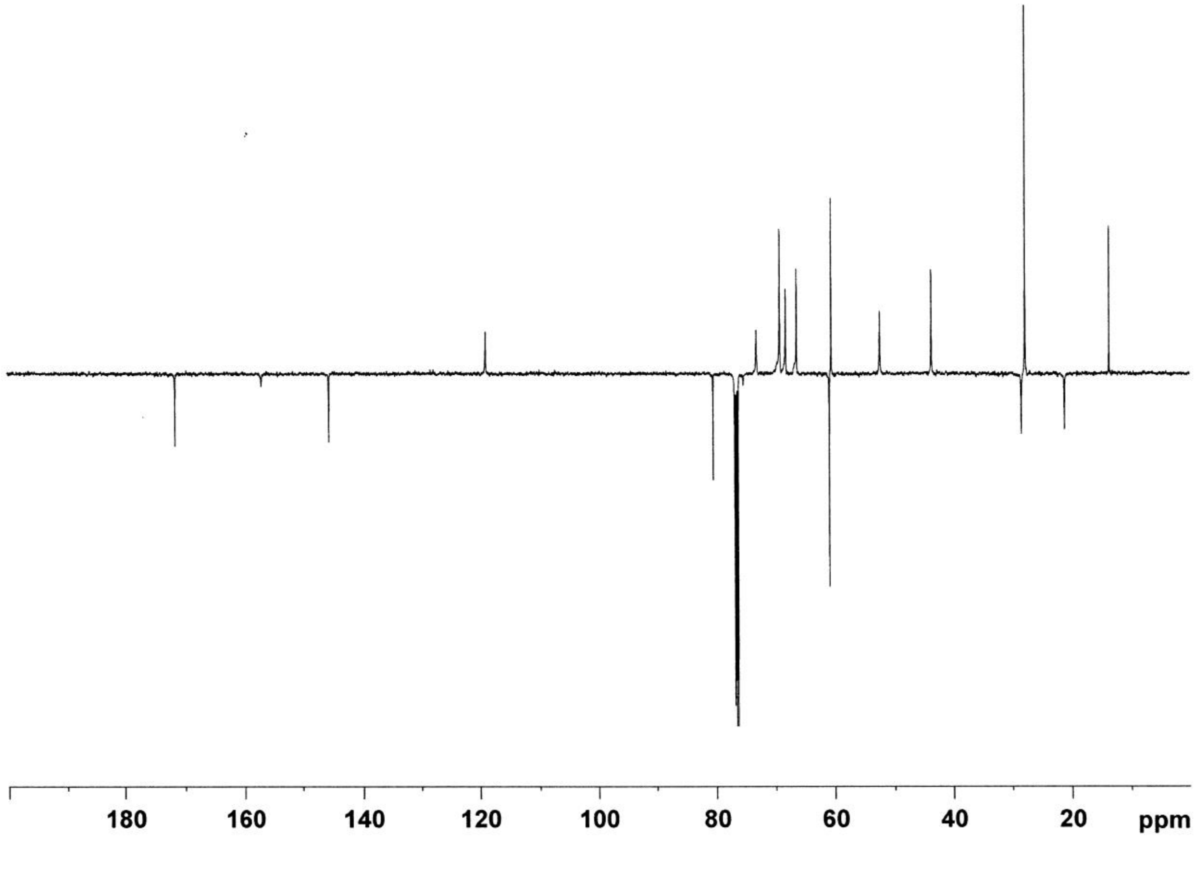

2.12. Triazole 26 ^{1}H and ^{13}C NMR in $CDCl_3$:

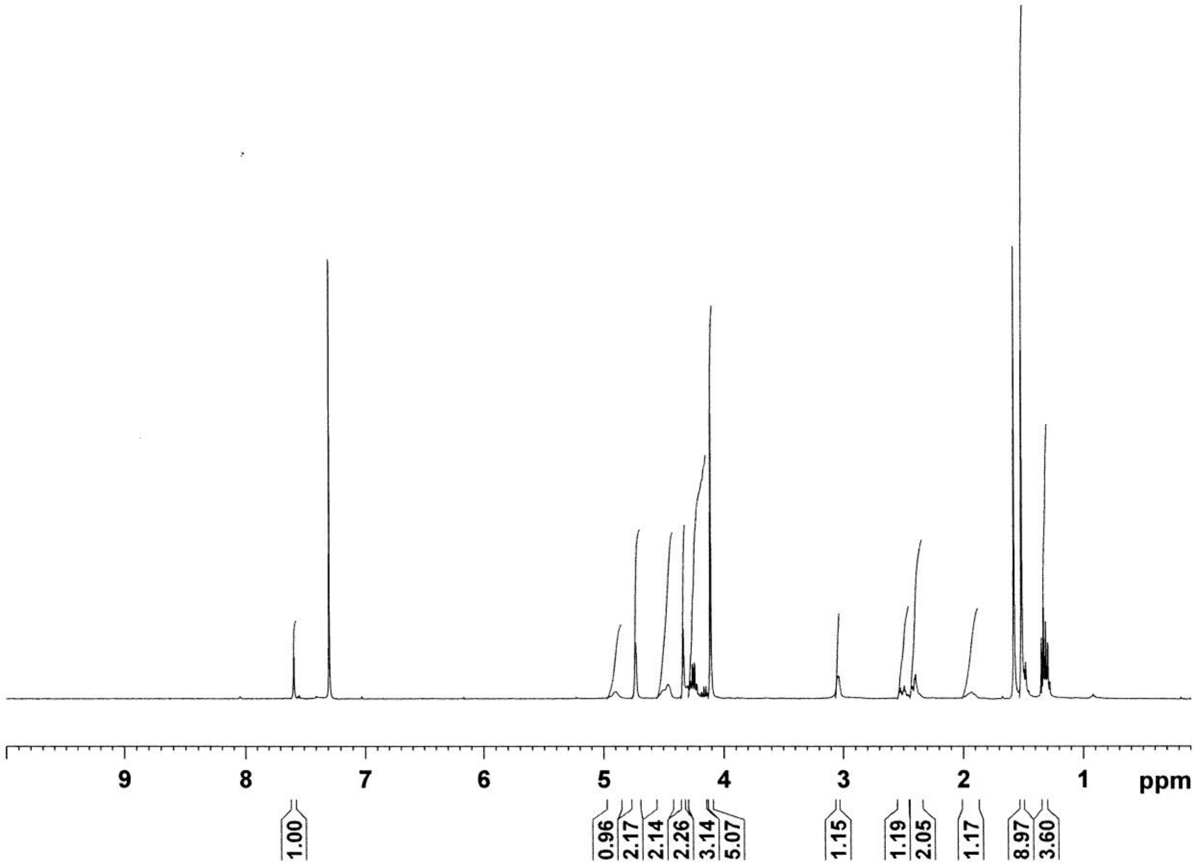

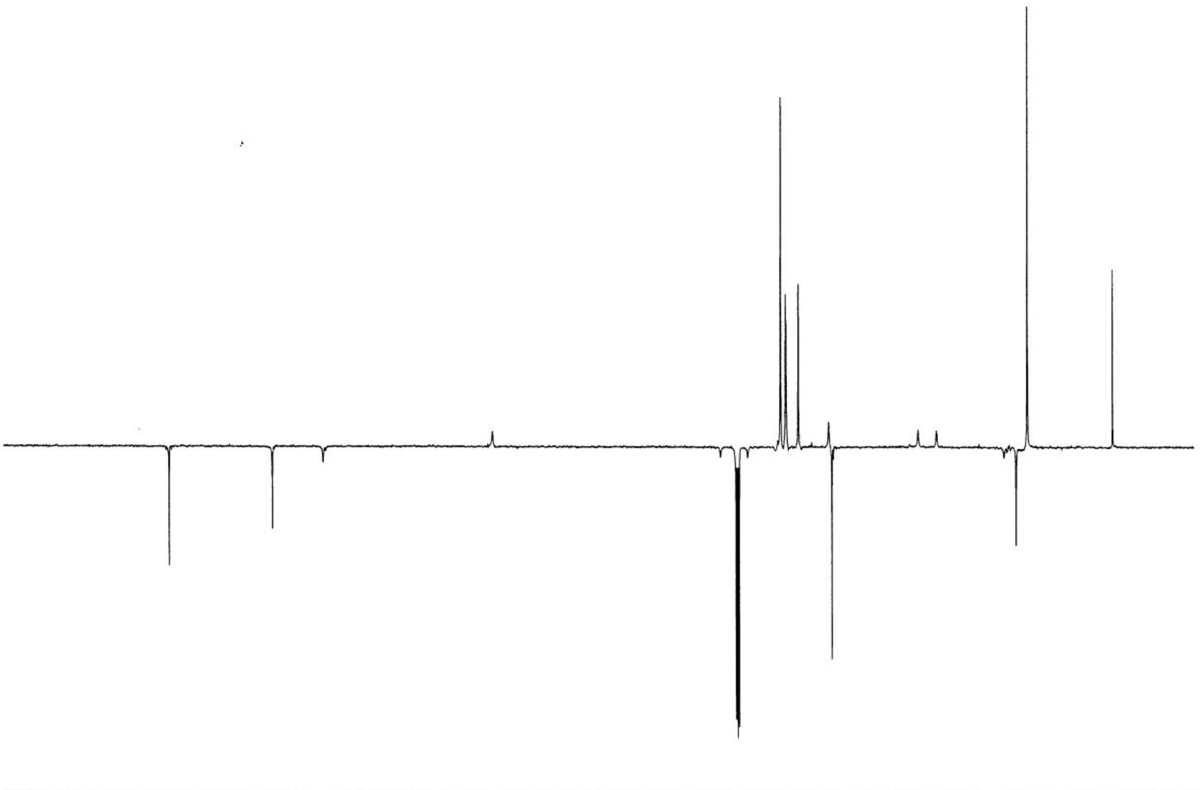

Supplement: File 1 — Detailed analytical data of the prepared compounds and a collection of NMR spectra. [file Beilstein_J_Org_Chem-09-1508-s001.pdf]
